# Genetically engineering self-organization of human pluripotent stem cells into a liver bud-like tissue using Gata6

**DOI:** 10.1038/ncomms10243

**Published:** 2016-01-06

**Authors:** Patrick Guye, Mohammad R. Ebrahimkhani, Nathan Kipniss, Jeremy J. Velazquez, Eldi Schoenfeld, Samira Kiani, Linda G. Griffith, Ron Weiss

**Affiliations:** 1 Department of Biological Engineering, Massachusetts Institute of Technology (MIT), Cambridge, Massachusetts 02139, USA; 2 MIT Emergent Behaviors of Integrated Cellular Systems (EBICS) Center, Cambridge, Massachusetts 02139, USA; 3 Synthetic Biology Center, MIT, Cambridge, Massachusetts 02139, USA

## Abstract

Human induced pluripotent stem cells (hiPSCs) have potential for personalized and regenerative medicine. While most of the methods using these cells have focused on deriving homogenous populations of specialized cells, there has been modest success in producing hiPSC-derived organotypic tissues or organoids. Here we present a novel approach for generating and then co-differentiating hiPSC-derived progenitors. With a genetically engineered pulse of GATA-binding protein 6 (GATA6) expression, we initiate rapid emergence of all three germ layers as a complex function of GATA6 expression levels and tissue context. Within 2 weeks we obtain a complex tissue that recapitulates early developmental processes and exhibits a liver bud-like phenotype, including haematopoietic and stromal cells as well as a neuronal niche. Collectively, our approach demonstrates derivation of complex tissues from hiPSCs using a single autologous hiPSCs as source and generates a range of stromal cells that co-develop with parenchymal cells to form tissues.

## Abstract (web summary)

There has been limited success in generating tissues from human induced pluripotent stem cells (hiPSCs). Here, the authors genetically engineer expression of the transcription factor Gata6 in a single isogenic hiPSC population resulting in complex tissue structures that exhibit liver bud-like properties.

## Graphical abstract

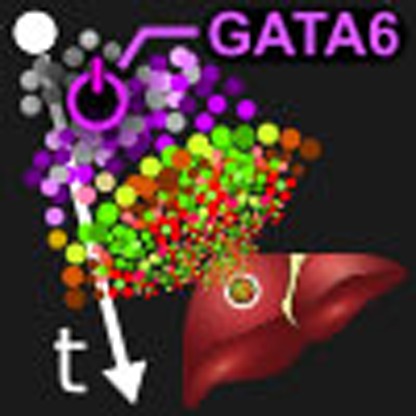

Development of complex multicellular systems based on human embryonic stem cells and human induced pluripotent stem cells (hiPSCs)^1^,^2^ is an emerging area of research exemplified by remarkable demonstrations of optic cup and brain organoid formation^3^,^4^. Another recent study combined hiPSC-derived hepatocyte-like cells with endothelial and mesenchymal cells isolated from umbilical cords to generate a liver bud tissue with some basic functionality *in vivo*^5^. These findings highlight the importance of self-organization and emergence, two inherent capabilities of hES and hiPSCs. Yet, these studies focus on deriving structures from a single germ layer (optic cup and brain organoid) or mixing cells isolated from multiple/postnatal individuals (liver bud). In contrast, organs *in vivo* comprise cells originating from more than one germ layer and arise in embryogenesis by having various progenitor cell types co-develop. Furthermore, tissues developed from autologous, singly derived hiPSCs are likely to be most amenable for translation to practical applications^6^. In addition to the envisioned therapeutic application of organoids, a pressing need exists for more predictive *in vitro* human tissue models for developing effective drug screens of efficacy and safety in humans. Both regenerative medicine efforts and *in vitro* organ models depend on robust protocols to grow organotypic tissues. Therefore, we set out to genetically engineer differentiation of a single-cell population to the three germ layers, with a focus on transcription factors that guide cell fate towards endodermal and mesodermal lineages. We surmised that ectodermal fate could be obtained without direct engineering, as it is the default pathway^7^. We decided to investigate the transcription factor GATA6, since it is involved in a wide range of functions including segregation of the blastocyst's inner cell mass into epiblast and primitive endoderm based on Nanog versus Gata6 levels^8^,^9^. Gata6 is also involved in subsequent gastrulation, mesoderm specification, cardiac development, lung endoderm branching, mesenchymal to epithelial transitions and organogenesis of pancreas, gut and liver, among others^10^,^11^. Ectopic overexpression of Gata6 in mouse embryonic stem cells induces visceral endoderm, upregulates bone morphogenetic protein 2 expression and promotes cell survival^12^. It was demonstrated that visceral endoderm intercalates with definitive endoderm and contributes significantly to the gut tube in mice^13^. During liver development, GATA6 is expressed in several tissues that influence development of the liver (for example, cardiac mesoderm and septum transversum mesenchyme) and maintains the expression of growth factors such as BMP4 (ref. 14). Gata6 also regulates hepatic fate by acting upstream of genes such as hepatocyte nuclear factor 4 (HNF4). But while Gata6 has an array of functions in organogenesis, analysis of rescued Gata6^−/−^ embryos shows that the development of many tissues and organs occurs normally, whereas liver development arrests shortly after generation of the primary hepatic bud^11^.

In this study, by engineering a wide range of Gata6 expression levels in a pluripotent cell population, we directed their differentiation into a heterogeneous tissue and detected a liver bud-like structure containing stromal cells, vascular tube-like structures and haematopoiesis-like processes.

## Results

### Gata6-induced symmetry breaking in hiPSCs

**Figure 1:**
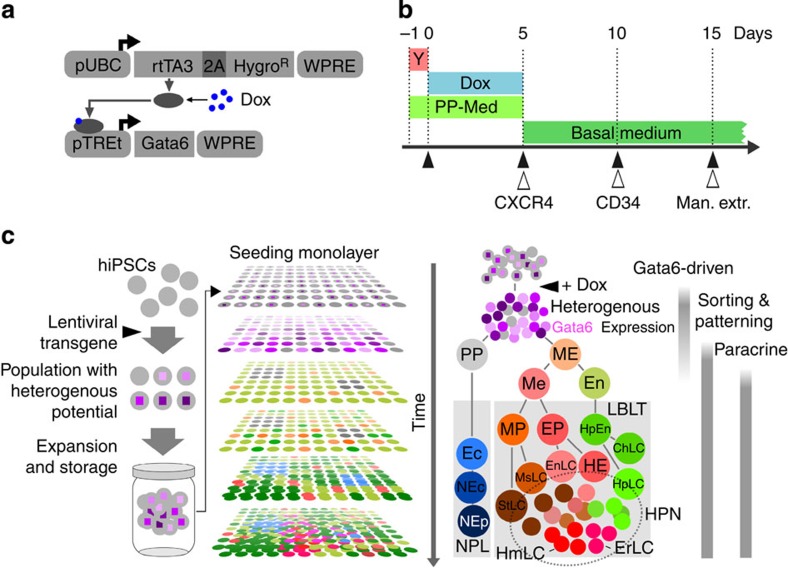
System overview. (**a**) Lentiviral constructs used to generate stable cells lines. (**b**) General timeline for experiments, media conditions and cell extractions for exome microarray analysis. Y, rock-inhibitor; Dox, doxycycline; PP-Med., pluripotency supporting medium. Basal medium: medium without additional growth factors or serum. Filled arrows: RNA isolation and microarray analysis from total cells. Open arrows: RNA isolation and microarray analysis from enriched cells (CXCR4, CD34: enrichment using MACS beads; man. extr., manual extraction of early neuronal cell clusters). (**c**) Process overview (left) and a model of cell types generated during experiment (right). hiPSC containing an inducible *GATA6* transgene are seeded in a monolayer. Transgene expression is then triggered with a small inducer molecule (Dox) causing the cells to co-differentiate. Starting with an undifferentiated monolayer of hiPSCs, we obtain complex tissue after 15 days. LBLT, liver bud-like tissue; NPL, neuronal progenitor-like; HPN, haematopoietic niche. HPN includes all cellular components developed within the system, which directly or indirectly support emergence of haematopoietic-like processes. For more information refer to the text. ME, mesendoderm, PP: pluripotent cells (expressing pluripotency markers, not induced to mesendoderm), En, endoderm; Me, mesoderm; Ec, ectoderm; HpEn, hepatic endoderm; EP, endothelial progenitors; MP, mesenchymal progenitors; Ec, ectoderm; NEc, neurectoderm; HpLC, hepatocyte-like cells; ChLC, cholangiocyte-like cells; EnLC, endothelial-like cells; HE, haemangioblast-like cells; ErLC, erythrocyte-like cells; HmLC, haematopoietic progenitor-like cells; StLC, stellate-like cells; MsLC, mesenchyme/pericyte-like cells; NEp, neuronal progenitors.

We hypothesized that GATA6 might not only be a good candidate to regulate early events (inner cell mass segregation, germ layer commitments), but also to control later differentiation processes in more specialized progenitor cells depending on GATA6 expression levels and tissue context. Our experimental approach is outlined in Fig. 1. At the core of our genetic manipulation lies a small gene circuit delivered by means of lentivirus, enabling small-molecule (Doxycycline, Dox)-induced ectopic expression of Gata6-encoding transgenes (for example, *Gata6*, *GATA6-2A-EGFP* and *GATA6-HA*) (Fig. 1a). As a function of the lentiviral transduction, cells receive different copy number of the transgene and exhibit a wide range of protein expression levels. Figure 1b shows the experimental timeline and Fig. 1c shows the experimental set-up and an overview of the cell types generated in the process. As a preview for results presented throughout this manuscript, the temporal expression pattern of key cellular markers we observed during the development of our complex tissue is depicted in Supplementary Fig. 1.

**Figure 2:**
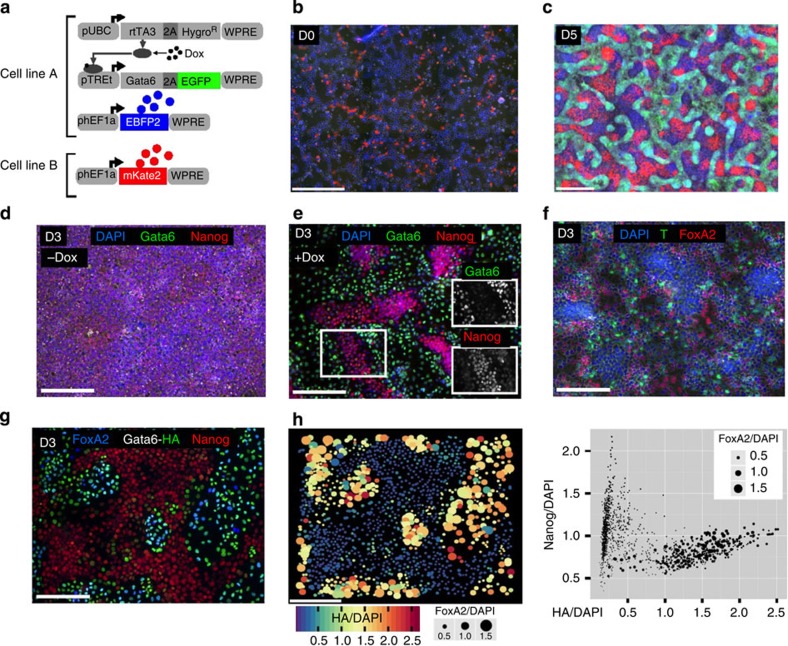
Ectopic expression of Gata6 in hiPSCs induces a heterogeneous multi population niche including CXCR4^+^ definitive endoderm. (**a**) Cell line A expresses EBFP2 (blue fluorescent protein) constitutively and GATA6-2A-EGFP (GATA6 and green fluorescent protein) in a Dox-inducible fashion. Cell line B expresses mKate2 (red fluorescent colour), constitutively. (**b**) Cell line A and B were mixed and seeded at a 9:1 ratio and induced with Dox (1,000 ng ml^−1^) (**c**). By day 5, the two cell lines proliferate and segregate to distinct high EGFP^+^(also EBFP^+^) and mKate2^+^ subpopulations. When the blue cells express high EGFP (high GATA6), they look blue and green. If they express low amounts of EGFP they look blue (very weakly green) and stay together with mKate^+^ population. (**d**) Undifferentiated uninduced hiPSCs at day 3 are enriched in Nanog. (**e**) Dox-induced ectopic expression of lentivirally delivered *GATA6* leads to segregation into GATA6^+^ or Nanog^+^ subpopulations. hiPSCs were seeded as single-cell suspension(+Dox, 1,000 ng ml^−1^). Insets show that Nanog^+^ are weakly GATA6^+^. (**f**) Brachyury^+^ (also known as T) cells are interspersed with FoxA2^+^ endodermal cells at day 3 for a Dox-induced experiment. (**g**) Immunostaining for HA, FoxA2 and Nanog. (**h**) Analysis of the immunostaining: GATA6-HA induces endoderm, but is suppressed in the Nanog^+^ cluster. Cells with low Nanog levels require less GATA6 (HA epitope) to differentiate into endoderm (FoxA2). Scale bars, 200 μm.

We first engineered two hiPS cell lines, one with and one without exogenous *GATA6*, induced with Dox, and observed spatial segregation of the populations (Fig. 2a–c, Supplementary Fig. 2). By starting with various ratios of the two cell lines, we demonstrate control over the generation of these clusters, yielding distinct immunostaining regions for GATA6 and NANOG (Supplementary Fig. 2a). Interestingly, in a separate experiment, we observed that culturing only one cell population that ectopically expresses GATA6 at a wide range of levels across the population is sufficient to induce segregation into an epithelial GATA6-positive endodermal subpopulation and compact clusters of an octamer-binding transcription factor 4 (OCT4) and NANOG-positive pluripotent subpopulations (Fig. 2d–f, Supplementary Figs 2b,3 and Supplementary Movie 1). Varying the Dox concentration also enabled us to fine-tune the resulting subpopulations (Supplementary Fig. 2b). Under full Dox induction, we observed markers for endoderm (forkhead box A2 (FOXA2), sex-determining region Y-box 17 (SOX17); Fig. 2f,g; Supplementary Figs 2 and 3) and mesoderm (Brachyury, also known as T) by day 3 (Fig. 2f). A similar population segregation took place when we co-translationally coupled GATA6 to a puromycin resistance marker (*GATA6-2A-puromycin*) and selected for resistant cells (Supplementary Fig. 3c,d), implying that resistance to the antibiotic via GATA6-2A-puromycin expression occurs at a different (apparently lower) threshold than GATA6-induced differentiation. In cell lines expressing GATA6 or HA epitope-tagged GATA6 (GATA6-HA), GATA6 and HA are readily detectable in the endodermal subpopulation, and also within the pluripotent subpopulation, albeit at lower levels (Fig. 2e,g,h, Supplementary Fig. 4). A sharp transition in a scatter plot of GATA6-HA versus FOXA2 levels suggests that GATA6 expression must reach a defined threshold for FOXA2 to be expressed (Supplementary Fig. 4b). These observations suggest two mechanisms in action: induction of an endodermal phenotype as a function of relatively high GATA6 levels and location-dependent determination of endodermal versus pluripotent phenotype for relatively low GATA6 levels. Cells expressing high levels of GATA6 readily localize to the endodermal subpopulation and acquire an endodermal phenotype. Low GATA6-expressing cells acquire an endodermal phenotype if they are located within the endoderm subpopulation, but do not acquire an endodermal fate if they are located within the high NANOG-expressing sub-pluripotent population (Fig. 2g,h and Supplementary Fig. 4)^15^. These observations imply a process that functions as a cell fate decision switch integrating cell-autonomous (GATA6 levels) and non-autonomous (cell types nearby) information^9^.

### Formation of a CXCR4^+^ cell population

**Figure 3:**
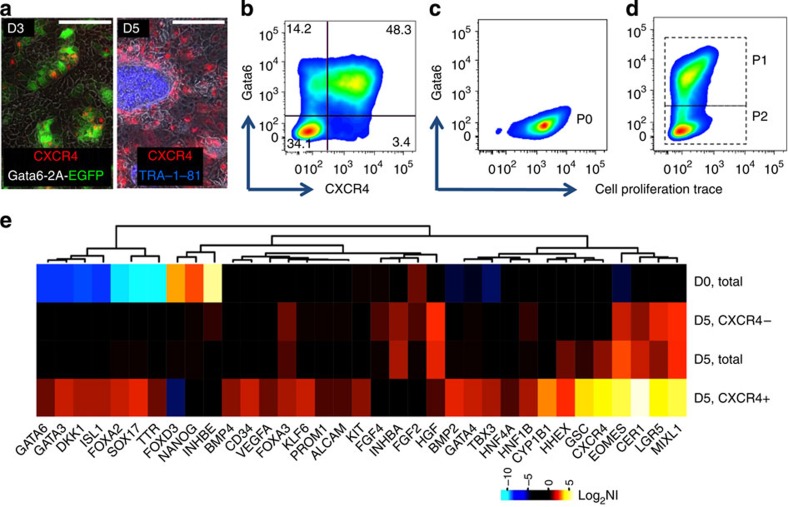
Characterization of CXCR4^+^ cells. (**a**) GATA6-expressing cells upregulate expression of CXCR4 (left). CXCR4-expressing cells surround TRA-1-81 compact cellular clusters at day 5 (right). TRA-1-81 recognizes hiPSC compact clusters at undifferentiated state. Scale bars, 200 μm. (**b**) Single-cell analysis of Dox-induced cells (1,000 ng ml^−1^) at day 5 shows ∼93% of CXCR4-expressing cells are also GATA6^+^. (**c**) CellTrace fluorescent stain labelled hiPSCs on day 0 before Dox (P_0_). The cells are transduced with Dox-inducible *GATA6-2*A*-EGFP* vector. (**d**) After 4 days of Dox (1,000 ng ml^−1^) treatment, *GATA6*-induced cells express EGFP (P_1_), proliferate and dilute the fluorescent dye through subsequent divisions to levels comparable to GATA6 non-expressing cells (P_2_) (**e**) CXCR4^+^ transcriptional profiling, D, Day of the experiment (D0=Addition of Dox to the cell culture medium). Log_2_ NI, log 2 normalized intensities. Scale bars, 200 μm.

To further characterize the specific subpopulations within our system, we studied surface markers, proliferation rate and performed cell isolation and downstream analysis. The surface marker CXC chemokine receptor 4 (CXCR4) appears on day 3 in GATA6-expressing cells and increases between days 3 and 5 (Fig. 3a). To further characterize the cultures, we took advantage of our hiPS cell line engineered with Dox-inducible *GATA6-2A-EGFP* transgene. In these cells, EGFP level can be quantified as a surrogate for GATA6 expression. Flow cytometry analysis after 5 days of Dox treatment shows a wide range of GATA6 expression across the population (Fig. 3b). On day 5 of differentiation, ∼77% of GATA6^+^ cells are CXCR4^+^ and ∼93% of the CXCR4^+^ subpopulation express *GATA6* transgene. Both the GATA6^+^ and GATA6^-^ subpopulations proliferate in the cultures at a comparable rate after 4 days (Fig. 3b–d). While CXCR4 has previously been associated with definitive endoderm and is still being used in many studies for this purpose, it became clear in recent years that it is also expressed on the surface of other cell types (visceral endoderm in mice, mesendoderm and trophoblasts)^16^. Species-specific divergences in early development may also explain to some extent the discrepancy in the literature^17^. In our system, CXCR4^+^ cells are found in higher density in the leading edges of the endoderm layer presumably by migrating to these areas (Supplementary Fig. 5a). We isolated CXCR4^+^ cells on day 5 using MACS beads (Miltenyi Biotech) (Supplementary Fig. 5b–d) and performed transcriptional profiling using exome microarrays. Markers enriched in day 5 CXCR4^+^ cells include primitive streak, mesendoderm, definitive endoderm, foregut and initial markers of hepatic endoderm (Fig. 3e, Supplementary Fig. 6a,b). This profile might represent asynchronously developing subpopulations, and we cannot exclude the presence of some primitive or visceral endoderm cells. The surface marker CD34 is also enriched in CXCR4^+^ cells (microarray data Fig. 3e). Markers depleted in the day 5 CXCR4^+^ subpopulation include FGF2, FGF4 and HGF, indicating that these important factors for hepatocyte specification and expansion are provided by the CXCR4^−^ fraction (Supplementary Fig. 6a). However, factors such as bone morphogenetic protein 2 (BMP2), BMP4 and vascular endothelial growth factor A (VEGFA) are enriched in CXCR4^+^ (Fig. 3e, Supplementary Fig. 6a). Heatmap clustering of gene expression data for markers specific for endoderm, mesoderm and ectoderm shows that while endodermal genes are enriched in CXCR4^+^ cells, mesodermal-associated genes are distributed in both CXCR4^+^ and CXCR4^−^ groups and ectodermal genes are upregulated in CXCR4^−^ cells (Supplementary Fig. 6b).

### Development of different fetal liver cell populations

**Figure 4:**
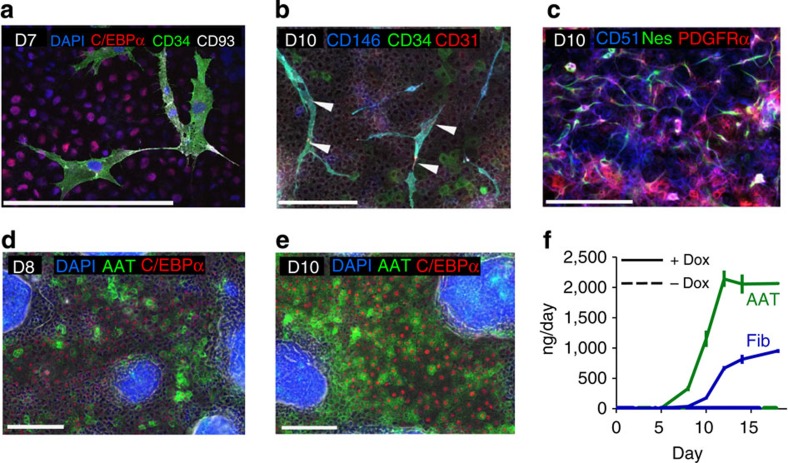
Development of endothelial and mesenchymal like cells and maturation of hepatic endoderm. (**a**) CD34 and CD93 were detected in endothelial progenitor-like cells on day 7. CEBPα^+^marks the hepatic endoderm in the background (scale bars, 200 μm). (**b**) Development of endothelial-like cells (arrows indicate CD31). CD34 is present in the hepatocyte-like cell fraction (CEBPα^+^, CD146^−^) and in the endothelial-like cells (CEBPα^−^, CD146^+^). (**c**) CD51^+^, NES^+^, PDGFRα^+^ mesenchymal stem cell-like cells develop in conjunction with hepatoblasts. (**d**) AAT and CEBPα on day 8 and (**e**) 10 show maturation of hepatic endoderm. (**f**) Upregulation of AAT and fibrinogen (Fib) synthesis (ELISA). Data are mean±s.e.m., *n*=3 per group. Scale bars: 200 μm.

**Figure 5:**
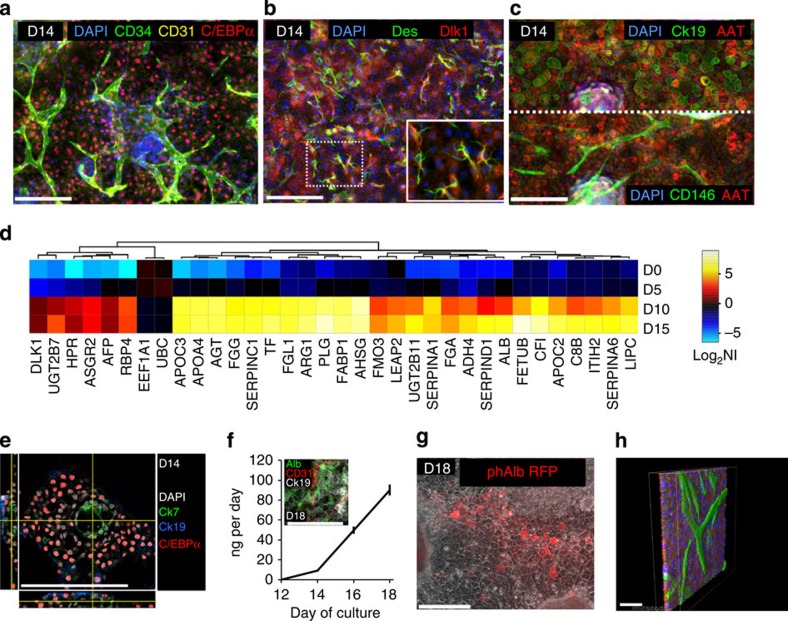
Development of fetal liver parenchymal and non-parenchymal cells. (**a**) CEBPα^+^ hepatocyte-like cells and CD31^+^ CD34^+^ vascular networks on day 14. (**b**) DES^+^ stellate-like cells (**c**) CK19^+^ AAT^+^ hepatocyte-like cells with CD146^+^ vascular-like structures. Scale bar, 200 μm. (**d**) Heatmap shows upregulation of hepatic genes between days 5 and 10. (UBC and EEF1A1 as used as control/housekeeping genes). (**e**) CK7^+^ bile duct-like channels develop within fetal hepatocyte-like cells; xz and yz slices on the left and bottom. Scale bar, 200 μm. (**f**) Further maturation of liver-like phenotype as evidenced by increasing albumin production (ELISA) and (**g**) albumin^+^ cells. Data are mean±s.e.m., *n*=3 per group. phAlb_mKate2 is a lentivirally integrated reporter where a short human albumin promoter drives expression of a red fluorescent protein (mKate2). Scale bar, 200 μm. (**h**) Confocal imaging and three-dimensional reconstruction of AAT^+^ hepatocyte-like cells (red) with an apical layer of CD34^+^ endothelial-like cells (green). Scale bar, 50 μm.

After day 5 of culture, we detected markers associated with cell types that are normally found in the developing liver bud. For instance, CD34^+^ cells start to emerge on days 6–7 (CD34^+^, CD146^+^, T-cell acute leukaemia protein 1^+^ (TAL1^+^), CD93^+^, fetal liver kinase 1^+^ (FLK1^+^), CCAAT/enhancer binding protein α^+^ (CEBPα^+^)) expressing markers typical for haemangioblasts and endothelial progenitors^18^ (Fig. 4a,b and Supplementary Fig. 7a–d). Cells expressing nestin (NES), CD51 and platelet-derived growth factor α (PDGFRα), markers of mesenchymal precursors associated with enhanced liver maturation, as well as expansion of haematopoietic progenitors^19^,^20^, develop within the endodermal layer (Fig. 4c). Intracellular CEBPα, secreted alpha-1 antitrypsin (AAT) and fibrinogen, three important hepatic associated proteins, strongly increase in expression between days 8 and 10 as measured in the supernatant by means of enzyme-linked immunosorbent assay (ELISA; AAT, fibrinogen) and with immunostainings (CEBPα, AAT; Fig. 4d–f), suggesting further maturation of hepatic endoderm. CD34^+^, CD146^+^ cells additionally acquire CD31 between days 7 and 10 and become endothelial-like cells while a group of CD34^+^ cells remains CD146^−^ with an endodermal morphology (Fig. 4a,b, Supplementary Fig. 7a–c). The CD34^+^ CD31^+^ cells integrate and form a vascular-like network by day 14 (Fig. 5a). The percent of HNF4α^+^ cells increases between days 6 and 14 and fetal hepatocyte-like cells also express the markers cytokeratin 19 (CK19), delta-like homologue 1 (DLK1), CEBPα, leucine-rich repeat-containing G protein coupled receptor 5 (LGR5) and AAT between days 10 and 14. Subpopulations of these cells are also epithelial cell adhesion molecule (EpCAM)^+^ or CD133^+^ (Fig. 5a–c, Supplementary Fig. 7e–j)^21^,^22^. In addition, the mean percentages of tissue area covered by AAT^+^ and CD34^+^ cells are 85.6 and 17.1%, respectively (Supplementary Fig. 7k). Desmin (DES)+cells with a typical stellate-like cell morphology appear from the NES^+^ population around day 10, and are interspersed with the hepatocyte-like cells (Fig. 5b, Supplementary Fig. 8a,b). Treatment with Axitinib (a small molecule tyrosine kinase inhibitor targeting VEGFR-1/-2/-3, PDGFR and c-Kit) starting on day 5 inhibits later emergence of CD34^+^ endothelial-like cells but not of CD34^+^ endodermal-like cells (Supplementary Fig. 8c). Some NES^+^ cells are also found in close association with CD34^+^ endothelial-like cells in a similar fashion to pericytes associating with blood vessels (Supplementary Figs 9 and 10)^23^.

Transcriptional analysis suggests induction of many genes associated with hepatic fate (Fig. 5d). Gene enrichment analysis also shows over-representation for liver-associated pathways including the complement cascade, a set of proteins synthesized by the liver that play an important role in the innate immune system (Supplementary Tables 1 and 2). Circular ducts containing cholangiocyte-like cells (CK7^+^ and aquaporin (AQP1^+^)) also develop within the fetal hepatocyte-like cell layer (Fig. 5e, Supplementary Fig. 11). Albumin protein increases steadily after day 12, indicating further maturation of the hepatic tissue and is also evident by cellular red fluorescence generated from lentivirally integrated reporter encoding a short human albumin promoter that drives expression of a red fluorescent protein (mkate2; Fig. 5f,g). Additionally, modulation of Dox-induced GATA6 expression can regulate hepatic fate differentiation, as evidenced by distinct fibrinogen levels produced in the media on day 14 (Supplementary Fig. 12a–d). In the absence of Dox, cells do not generate endoderm or mesoderm. The developed tissue is organized in layers of cells with a thickness ranging from one to a few cells (Fig. 5e,h and Supplementary Fig. 13, Supplementary movie 2).

### The emergence of haematopoietic properties

**Figure 6:**
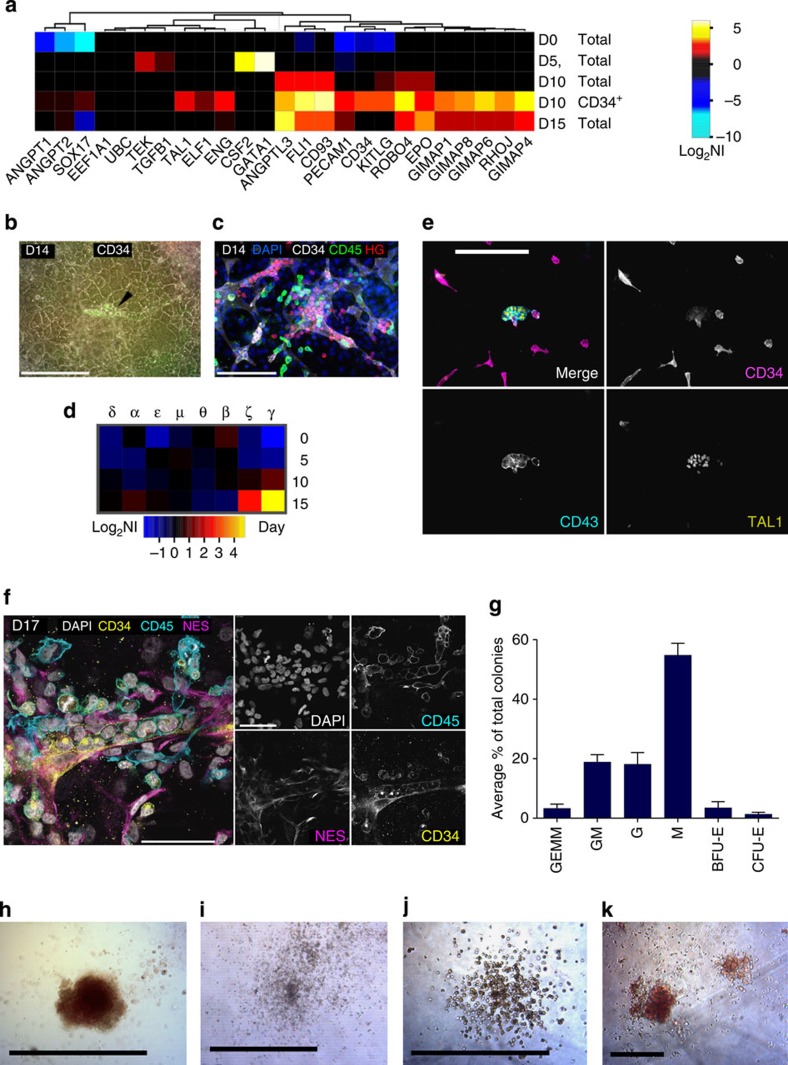
The emergence of fetal haematopoiesis after day 14. (**a**) Heatmap shows temporal upregulation of markers associated with endothelial and haematopoietic cells in CD34^+^ isolated cells on day 10. (**b**) Black arrow: endothelial-like cell tube embedded in hepatocyte-like cells and filled with CD34^+^ cells. (**c**) Immunostaining shows expression of CD45 and HG. HG, pan-haemoglobin. Scale bar, 200 μm. (**d**) Microarray analysis shows haemoglobin γ expression is strongly upregulated by day 15. The Greek letters are representative of different types of globin chain of human haemoglobin expressed at different developmental stages^25^. Scale bar, 200 μm. (**e**) Development of TAL1^+^ CD43^+^ cells among CD34+ population on day 8. (**f**) Left: close-up of a CD34^+^ tube-like structure covered by pericyte-like cells (NES^+^) that contains CD45^+^ haematopoietic-like cells on day 17. Scale bar, 200 μm. (**g**) Multipotency of isolated CD34^+^ population assessed by the Methocult CFU assay, showing generation of (**h**) erythrocyte (BFU-E) (**i**) granulocyte, macrophage (GM) (**j**) macrophage (M), and (**k**) granulocyte, erythrocyte, monocyte, megakaryocyte (GEMM) multicellular colonies. Scale bars in **h**–**k**, 100 μm. Data are mean±s.e.m. and representative of at least three cultures per group.

Microarray data for day 5 cells shows upregulation of growth factors and transcription factors associated with haemangioblast induction including vascular endothelial growth factor–A (VEGF-A; CXCR4^+^ cells, Fig. 3e), colony stimulating factor 2 (also known as granulocyte, macrophage–colony stimulating factor), TEK (also known as TIE2), GATA1 and transforming growth factor β1 (TGFβ1; total cells, Fig. 6a). Isolated CD34^+^ cells on day 10 exhibit higher expression levels for genes associated with endothelial and haematopoietic cells (Fig. 6a). Around day 14, CD34^+^ endothelial tubes constrict and bud off small spherical cells expressing CD45 or haemoglobin (Fig. 6b,c). Haemoglobin gamma is most prominently upregulated on day 15 (Fig. 6d), indicating definitive fetal erythropoiesis^24^,^25^. The embryonic liver bud is an important source of haematopoietic progenitor cells^26^. Primary DLK1^+^ hepatoblasts isolated from mice fetal liver are known to secrete cytokines promoting haematopoiesis/erythropoiesis^27^, and CD34^+^ cells from midgestation human fetal liver can efficiently reconstitute human haematopoietic cells as well as hepatocyte-like cells^22^. NES^+^ mesenchymal stromal cells and stellate cells were identified as regulator of haematopoietic processes in the fetal liver^28^,^29^,^30^. In fact, the developed tissue in this study contains DLK1^+^ hepatoblast-like cells, DES^+^ DLK1^+^ stellate-like cells, NES^+^ pericyte-like cells and CD34^+^ endothelial-like cells that may collectively act as components of the haematopoietic niche in the organoid.

Early progenitor commitment to haematopoietic tissue development can be recognized by leukosialin (CD43) expression^31^. We therefore, evaluated the presence of CD43 at day 8 when CD34^+^ cells were beginning to emerge. Within the CD34^+^ CD43^+^ subpopulation, we detected co-expression of TAL1, a protein that regulates haematopoietic specification (Fig. 6e). By day 17 we observed expansion of CD34^+^ subpopulation, leading to CD34^+^CD43^-^ vascular networks with patches of CD34^+^CD43^+^ cells that are associated with NES^+^ mesenchymal cells (Fig. 6f and supplementary Fig. 10a). We detected endothelial-like cells densely filled with small spherical cells expressing gradients of CD45 versus CD34 (Fig. 6f: high CD34, low CD45 to low CD34, high CD45). Nevertheless, we cannot determine whether the cells are generated from the endothelial-like cells (for example, hemogenic endothelium) and then downregulate CD34 while upregulating CD45, or if they are generated outside the endothelial-like cells and subsequently invade them to possibly to access the blood stream and then upregulate CD45. Next, we performed a functional *in vitro* assay (MethoCult) using the isolated CD34^+^ cells, which validated the presence of multipotent progenitors with an ability to undergo differentiation to erythroid and myeloid colonies. As expected, CD34^+^ cells were more potent with respect to haematopoietic progenitor capacity than CD34^−^ and total cell population (Fig. 6g–k and Supplementary Fig. 14a).

In the developing liver, Oncostatin M (OSM) is expressed in CD45^+^ haematopoietic cells, induces expression of liver-specific differentiation markers and stimulates several hepatic functions such as lipid and glycogen synthesis^32^. We assayed OSM in the collected media at several time points between days 16 and 30 and did not detect secreted OSM in the media (Supplementary Fig. 14b). Although current liver differentiation protocols based on sequential addition of cytokines normally administer OSM at ∼20 ng ml^−1^ to induce hepatocyte maturation^33^, it is possible that in our strategy multiple other redundant signalling pathways collectively drive liver differentiation in the developed tissue, compensating for the need for high concentration of a single factor that is used conventionally as a component of the differentiation medium. However, OSM signalling may be a potential candidate for future engineering to direct maturation of hepatocyte-like cells in this developed tissue. To investigate this question, we supplemented OSM in the media for a short period (days 24–29) and observed enhanced production of fibrinogen and AAT in the hepatic tissue that was subsequently attenuated on OSM removal (Supplementary Fig. 14c,d). It has been shown that OSM not only induces hepatic maturation but also downregulates fetal liver haematopoiesis^34^. Therefore, future studies should take into account that further promoting maturation to adult liver using this strategy may suppresses other emergent programmes^32^,^34^.

### Cell fate characterization in GATA6^+^ and WT cells

CD34^+^ cells are known to be developmentally associated with mesodermal germ layer. Therefore, we set out to further investigate their initial parental population, testing the hypothesis that the CD34^+^ population is generated from wild-type (WT) cells that are induced towards mesoderm fate by GATA6-driven endodermal cells. To study this, we used our hiPSC GATA6-2A-EGFP cells. Following Dox induction on day 2, we isolated the high GATA6-expressing population using fluorescence activated cell sorting, and cultured this (high) GATA6 population for an additional 14 days. We showed generation of CD34^+^ endothelial-like cells and vascular structures among AAT^+^ fetal hepatocyte-like cells (Supplementary Fig. 15a,b). In agreement with this observation, single cell analysis of GATA6-2A-EGFP cells on days 5 and 8 showed a growing population of CD34^+^ cells from both GATA6^+^ and CXCR4^+^ cells which was also supported by higher expression of CD34 transcript in CXCR4^+^ isolated cells when compared with the total population (Fig. 3e, Supplementary Fig. 15c–e). Thus, CD34^+^ cell fate selection is not exclusively dependent on the presence of an initial WT population. We then investigated whether silencing of *GATA6* transgene expression is a prerequisite step, allowing the cells to acquire a mesodermal CD34 fate when communicating with endodermal layer. To analyse this, we used our *GATA6-HA* engineered cell line where HA is stained as a surrogate marker of GATA6 expression. After development of the tissue, we re-administered Dox to evaluate activation of transgene expression in both hepatocyte-like and CD34+ endothelial-like cells. HA was observed in most hepatocyte-like cells and a subpopulation of endothelial-like cells (Supplementary Fig. 15f,g), indicating that GATA6 silencing is not necessary for acquisition of CD34 fate.

We then investigated whether the WT subpopulation contributes to other mesodermal components in our system. Specifically, we combined WT cells (marked by constitutive expression of mKate2) with a *GATA6* transduced population and monitored tissue development. Following expression of GATA6, WT cells initially sort out into compact red clusters of mKate2^+^ cells. Through the course of differentiation, we observed that some mKate2^+^ cells migrated out into the endodermal layers. We stained these cells for DES, NES and CD34 mesodermal markers and detected mesodermal fate differentiation in these migrated cells (Supplementary Fig. 16a–d), indicating that both GATA6^+^ and GATA6^−^ (WT) subpopulations contribute to mesoderm development in our system. Presumably additional regulatory elements such as the relative level of GATA6 in comparison to other transcription factors, kinetics of Gata6 expression and relative cell location collectively modulate mesodermal fate determination.

### Ectodermal cell fate and early neuronal cells

In addition to the liver phenotype discussed above, we observed markers associated with other phenotypes in the tissue. For example, alongside the mesendoderm-derived tissue, we discovered that the pluripotency and ectoderm marker OCT4 increases in intensity during the first five days (Supplementary Fig. 17a) in clusters of cells that initially maintain high NANOG expression (Fig. 2d,e,g). These cells subsequently acquire ectodermal markers on say 8 (AP2^+^, SOX10^+^) and later FOXG1 and sine oculis-related homobox 6 (SIX6) (Supplementary Fig. 17b), indicating maturation to neuronal progenitor-like and neural fold-like cells (Supplementary Note 1). While endoderm is involved in forebrain specification during embryonic development in vertebrates^35^, analysis of the extent to which ectodermal co-development with endodermal tissue is essential for induction of neuronal folds from hiPSCs is a topic for future investigation.

### Reproducible and stable tissue formation by Gata6 induction

Longer term observations until day 30 show stable tissue function as assessed by AAT and fibrinogen production (Supplementary Fig. 14c,d ; the blue line: OSM^−^ group). In addition, staining for hepatic and endothelial markers between days 18 and 22 reveals a maintained hepatocyte layer with vascular-like structures (Supplementary Fig. 18a–d). As shown in Supplementary Figs 1 and 9, Gata6 dosage controlled by Dox level as well as the initial viral transduction can regulate the development of liver bud-like phenotype. Notably, the tissue development in our system did not require addition of exogenous cytokines or growth factors to the cell culture medium beyond factors required for maintaining hiPSC pluripotency. While these pluripotency maintaining factors were only included during the first few days, it is possible that some of them (for example, FGF2 in mTeSR1) synergize with the pulse of Gata6 during the development of the tissue we obtained. Our approach also proved to be functional in all four hiPSC cells lines we tested: PGP1, PGP5, PGP9 and C1, confirming the reproducibility and robustness of the system in different cell lines (Supplementary Fig. 19a–c). In addition, using pure IMDM medium without serum or other additives still resulted in hybrid liver-bud-like structures and early neuronal tissues (Supplementary Fig. 19d).

## Discussion

Collectively, we generated fetal hepatocyte-, cholangiocyte-, endothelial-, haematopoietic-, stellate- and pericyte-like cells that co-develop and self-organize into a complex organoid. We also observed maturation of the ectodermal fraction towards a neuronal-like phenotype. Our study sets the stage for novel engineering approaches based on triggering co-differentiation events to reach a complex tissue with epithelial and stromal cell types. Here engineering heterogenic expression of *GATA6* generates a complex tissue that produces AAT (an important anti-neutrophil elastase) and fibrinogen (a key coagulation factor) in levels comparable to those generated by human cryopreserved hepatocytes, a gold standard cell type (Supplementary Fig. 20a,b). The tissue does not generate the same level of albumin production per hepatocyte-like cell when compared with cryopreserved hepatocytes (Supplementary Fig. 20c). This is expected, since the tissue exhibits characteristics consistent with fetal liver. Sorting for the high Gata6-expressing cells on day 2 and subsequent culturing resulted in large-scale tissues without patches of AAT^−^ CD34^−^ cells (ectodermal cell clusters) (Supplementary Fig. 21). This approach provides an important basis for strategies to enrich the cultures towards the liver type, if desired.

Our system has several advantages over embryoid bodies, which have been the method of choice for generating haematopoietic progenitor-like cells and other specialized cell types from hiPSCs in recent years. While intractable differentiation processes occur deep inside embryoid bodies, in our system the developmental processes are highly accessible and can be visualized, analysed and optimized easily without disrupting tissue and extracting specific cells. We do not rely on sequential administration of a complex cocktail of cytokines^36^. In fact, through co-differentiation of progenitor cells we generated niches demonstrating emergence of new cell types such as haematopoietic-like cells. Although the developed tissue does not have an intricate three-dimensional structure, it contains various cell types and exhibits phenotypes resembling key features of liver development including hepatic and haematopoietic functions. As our system generates all required specialized cells and cytokines in a self-contained fashion, manual intervention is unnecessary, greatly reducing experimental variability and costs. In contrast to embryoid bodies, our system permits more efficient and homogeneous exchange of oxygen, nutrients and cytokines as the cells develop and form a tissue on the surface of a cell culture dish. We also have not encountered any fragmentation issues that often plague embryoid body maintenance. While a recent study showed generation of a liver-like tissue by combining hiPSC-derived hepatocyte progenitors with human umbilical cord and mesenchymal stem cell lines^5^, in this study we generated many cell types associated with a liver bud from a single isogenic hiPSC population.

This study emphasizes the importance of co-differentiating complex populations of progenitors, recapitulating the intricate processes of embryogenesis, and setting into motion processes that lead to emergence of tissues and organs. Lentiviral gene delivery might not be optimal for therapeutic applications, although in this case it contributed to the wide range of GATA6 expression levels. In the future, it may be interesting to consider alternative expression methods that do not require foreign DNA (for example, RNA replicons or modified RNA) for driving strong transgene expression in hiPSCs^37^. The tissue we generated is embryonic in nature, and while further maturation *in* or *ex vivo* will be necessary to achieve the full functionality of an adult organ, this embryonic state provides us with highly valuable processes not available in the adult tissue (for example, haematopoiesis, specific progenitors). Our robust, self-organizing hiPSC-only approach has potential for developing improved tissue models (for example, for drug screening^38^) as well as for therapeutic applications (regenerative medicine) or as a model to study our own development.

## Methods

### Cell culture

The PGP1, PGP5 and PGP9 hiPSCs were a kind gift from George Church (Harvard University, USA) and can be obtained from Coriell (NJ, USA). The C1 hiPSC was a kind gift from Rudolf Jaenisch (MIT/Whitehead, USA). Cells were cultivated under sterile conditions in mTeSR-1 (Stem Cell Technologies, Vancouver) in a humidified incubator at 37 °C and 5% CO_2_. Tissue culture plates were coated for 1 h at room temperature with BD ES-qualified Matrigel (BD Biosciences) diluted according to the manufacturer's instructions in ice cold DMEM/F-12 with 15 mM HEPES medium (Stem Cell Technologies, Vancouver). Routine passaging was performed by incubating hiPSC's for 7 min in Dispase (1 ml per 10 cm^2^, Stem Cell Technologies, Vancouver) at 37 °C followed by three 2 ml washes in DMEM/F-12 medium and mechanical dissociation (#3010 cell scraper, Corning). Subsequently the cells were taken up in 5 ml of DMEM/F-12, centrifuged at 500 r.p.m. for 3 min and resuspended in mTeSR-1. Clump size was assessed by eye and if necessary a further reduction in size was performed by gently pipetting the suspension. Single-cell suspensions were generated by incubating hiPSC colonies for 5 min in Accutase (Stem Cell Technologies, Vancouver) at 37 °C, subsequently resuspending this single cell solution in 5 ml of DMEM/F-12 medium containing 10 μM Y-27632 dihydrochloride (Tocris Biosciences, UK) solubilized in cell culture grade DMSO (Sigma-Aldrich), centrifuging it at 500 r.p.m. for 3 min and resupending the pellet in mTeSR-1 or DMEM/F-12 supplemented with Y-27632 at a final concentration of 10 μM for counting.

In differentiation experiments, hiPSCs were seeded at a density of 25,000 cells per cm^2^ in mTeSR-1 supplemented with 10 μM Y-27632. The next day, the medium was changed to mTeSR-1 with 1000, ng ml^−1^ Dox and replaced daily for 5 days. Subsequently, a non-pluripotency supporting medium (APEL, Stem Cell Technologies, Vancouver) was used to grow the cells and replaced daily. Where a different Dox concentration is used, it has been specified in the manuscript text. During Oncostatin M dosing, cells were treated once per day with 20 ng ml^−1^ OSM from days 24to 29 and media was collected for ELISA assays. APEL contains human serum albumin. Therefore, when assaying for human albumin, F-12 media was used as non-pluripotency supporting medium and switched to William's E Medium with classical hepatocyte maintaining supplement (Life Technologies) consists of penicillin–streptomycin, insulin (6.25 μg ml^−1^), transferrin (6.25 μg ml^−1^), selenium complex (6.25 ng ml^−1^), Bovine serum albumin (1.25 mg ml^−1^), linoleic acid (5.35 μg ml^−1^), GlutaMAX (2 mM) and HEPES (15 mM) at day 12. To investigate silencing of *GATA6* transgene expression during hiPSCs differentiation to hepatocyte-like or CD34+ endothelial-like cells, hiPSCs transduced with *GATA6-HA* were used. Cells were redosed with Dox on day 17 for 2 days, before being fixed for staining of HA. To investigate whether the WT subpopulation contributes to mesodermal components in the developed organoids, WT cells (marked by constitutive expression of mKate2) and *GATA6* transduced hiPSC were combined (1:4 ratio), seeded and induced to differentiate. The developed tissues were fixed and stained on day 20.

For culturing cryopreserved human hepatocytes three cell lots (Hu8163, Hu1423 and Hu8160, from Life Technologies) were used. The cells were seeded in William's E Media containing classical hepatocyte seeding and maintaining supplements (Life Technologies) and overlaid with BD Matrigel according to the manufacturer's protocol.

### DNA constructs

Oligonucleotide sequences are listed in Supplementary Table 3. The UBC promoter was amplified from pFUW^39^ using oligos oPG106 and oPG107 and TOPO cloned into pENTR_L4R1 (Life Technologies), resulting in pENTR_L4_UBC_R1. pENTR_L4_TRET_R1, pENTR_L1_EGFP_L2, pENTR_L4_hEF1a_R1 and pENTR_L4_MCS_R1 are described elsewhere^40^. For constructing pENTR_L1_rtTA3-2A-Hygro_L2, rtTA3 was amplified using oPG106/107 and digested with BamHI/EcoRI. This fragment was ligated with a MfeI/NotI-cut PCR product encoding a 2A-hygromycin (amplified in two steps using oPG316a/oPG317 then oPG316b/oPG317) into a previously BamHI/NotI-cut pENTR_L1L2 (Life Technologies). pENTR_L1_hGata6_L2 was constructed by PCR-amplifying all six genomic exons in the human Gata6 gene from genomic DNA using the oligos pPG6371-6381 and assembling the parts in a single pot reaction into pENTR_L1L2 by means of the Golden Gate Reaction^41^. pENTR_L1_hGata6-HA_L2 was constructed by PCR-amplifying human *GATA6* from pENTR_L1_hGata6_L2 using oligos oPG_hG6_RegStartF and oPG_hG6_RegTermR and recombining the resulting PCR product into pDONR221P1P2 using the BP reaction (Life Technologies). pENTR_L1_mGata6_L2 was constructed by amplifying *Gata6* from mouse *Gata6* cDNA (kind gift of I. Lemischka, Mt. Sinai Medical School, NY) using oPG630/621 and recombining the resulting PCR product into pDONR221P1P2 using the BP reaction. pENTR_L1_hGata6-2A-Puro_L2 was constructed by PCR-amplifying Gata6 from pENTR_L1_hGata6_L2 using oPG6382/6383, 2A-Puromycin from AAVS1-SA-2A-puro-pA (Addgene Plasmid 22075) using oPG6385/6386 and cloning the products by means of a Golden Gate Reaction into pENTR_L1L2. pENTR_L1_hGata6-2A-EGFP_L2 was constructed by PCR-amplifying Gata6 from pENTR_L1_hGata6_L2 using oPG_hGX-2A-EGFP_G6-fwd/oPG_GX-2A-EGFP_G6-rev and EGFP from pFUW using oPG_hGX-2A-EGFP_EGFP-fwd/oPG_hGX-2A-EGFP_EGFP-rev and cloning the parts into pENTR_L1L2 using a Golden Gate Reaction. pENTR_L1_mKate2_L2 was constructed by means of gene synthesis on the template of mKate2 (Evrogen, Russia). pENTR_L4_hAlb_R1 was constructed by PCR-amplifying a promoter fragment spanning base pairs −1966 to +35 relative to the transcriptional initiation of the human albumin gene from genomic DNA using oPG5141/oPG5151, digesting the resulting fragment with XhoI/EcoRI and restriction enzyme cloning it into XhoI/EcoRI-cut pENTR_L4_MCS_R1. peNTR_L1_EBFP2_L2 was constructed by PCR-amplifying EBFP2 from pLV-EBFP2-nuc (Addgene Plasmid 36085) using oPG1162/oPG1163 and recombining the resulting PCR product into pDONR221P1P2 by means of the BP reaction (Life Technologies). pLV_Dest-R4R2 was constructed by PCR-amplifying the backbone of pFUW-OSKM (Addgene Plasmid 20328) with oPG240/oPG241, digesting the product with PacI and EcoRI and ligating it with a PacI/MfeI-cut PCR product amplified with oPG242/oPG243 from pLenti6/R4R2/V5-DEST (Life Technologies). pLV_UBC_rtTA3-2A-Hygro was constructed by recombining pENTR_L4_UBC_R1 with pENTR_L1_rtTA3-2A-Hygro_L2 into pLV_Dest-R4R2 using the LR Recombinase (Life Technologies). pLV_TRET_mGata6 was constructed by recombining pENTR_L4_TRET_R1 with pENTR_L1_mGata 6_L2 into pLV_Dest-R4R2 using the LR Recombinase. pLV_TRET_hGata6-HA was constructed by recombining pENTR_L4_TRET_R1 with pENTR_L1_hGata6-HA_L2 into pLV_Dest-R4R2 using the LR Recombinase. pLV_TRET_hGata6-2A-EGFP was constructed by recombining pENTR_L4_TRET_R1 with pENTR_L1_hGata6-2A-EGFP_L2 into pLV_Dest-R4R2 using the LR Recombinase, pLV_TRET_hGata6-2A-Puro was constructed by recombining pENTR_L4_TRET_R1 with pENTR_L1_hGata6-2A-Puro_L2 into pLV_Dest-R4R2 using the LR Recombinase. pLV_hAlb_mKate2 was constructed by recombining pENTR_L4_hAlb_R1 with pENTR_L1_mKate2_L2 into pLV_Dest-R4R2 using the LR Recombinase. pLV_hEF1a_mKate2 was constructed by recombining pENTR_L4_hEF1a_R1 with pENTR_L1_mKate2_L2 into pLV_Dest-R4R2 using the LR Recombinase. pLV_hEF1a_EBFP2 was constructed by recombining pENTR_L4_hEF1a_R1 with pENTR_L1_EBFP2_L2 into pLV_Dest-R4R2 using the LR Recombinase.

### Lentiviral production and transduction

HEK293FT cells (Life Technologies) were grown according to the manufacturer's indication in a humidified incubator at 37 °C with 5% CO_2_. The day before transfection we seeded 8 million HEK293FT cells on a Gelatine-coated 150 cm^2^ cell culture dish. On the day of transfection, we replaced the cell culture medium and co-transfected the cells with 15 μg pCMV-dR8.2 dvpr (Addgene Plasmid 8455), 3.75 μg pCMV-VSV-G (Addgene Plasmid 8454) and 11.25 μg of the plasmid to be packaged using Metafectene Pro (Biontex, Germany). Six hours post-transfection the medium was changed and 20 ml of fresh cell culture medium added to the cells. Two days later, supernatant was collected and stored at 4 °C. A measure of 20 ml of fresh cell culture medium was added to the cells. The next day, supernatant and the previous day's stored supernatant were pooled, filtered through a 0.45 μm low protein binding filter (Corning) and then further concentrated in an Amicon Ultra 15 filter columns (100 kDa cutoff, Millipore) at 4,000*g* to a final volume of 400 μl. The concentrated virus was then aliquoted and stored at −80 °C. hiPSCs were transduced as single-cell suspensions in Matrigel-coated six or twelve well cell culture plates in mTeSR-1 containing 10 μM Y-27632. The medium was changed the next day.

### Immunofluorescence

Cells were grown on Matrigel-coated glass coverslips and fixed for 20 min in 4% fixation buffer (BioLegend, USA) at room temperature. Coverslips were then washed three times in 250 μl PBS spotted on Parafilm M (Pechiney Plastic Packaging Company) followed by 15 min permeabilization in 100 μl of 0.2% Triton X-100 in PBS. Subsequently the coverslips were washed three times in 250 μl in PBS for 5 min and blocked for 20 min in 250 μl 4% normal donkey serum (Abcam, USA) in PBS. The incubation with the primary antibodies was performed for 1 h at room temperature in 25 μl of 4% normal donkey serum in PBS followed by three washes in 250 μl in PBS for 5 min. The incubation with the secondary antibodies was performed for 1 h at room temperature in 25 μl of 4% normal donkey serum in PBS followed by three washes in 250 μl in PBS for 5 min. Finally, the coverslips were mounted on microscopy glass slides using ProLong Gold antifade (Life Technologies, USA), cured overnight at room temperature and then sealed with nail polish. A list of all the antibodies used in this study is available in the Supplementary Information.

Primary antibodies: PAX7 (R&D MAB1675, 1:500), PROX1 (Abcam ab37128), SOX17 (R&D AF1924, 1:200), NANOG (Abcam ab80892, 1:200), CEBPA (R&D AF7094, 1:200), DES (Santa Cruz sc-7559, 1:200), DES (Santa Cruz sc14026, 1:200), AAT (R&D AF1268, 1:200), FOXG1 (Abcam ab18259, 1:500), SOX10 (Abcam ab155279, 1:200), DLK1 (Abcam ab89908, 1:200), TFAP2A (Abcam ab11828, 1:500), TFAP2A (Santa Cruz sc12726, 1:200), CD45 (Abcam ab33522, 1:200), NES (Santa Cruz sc21247, 1:200), KRT19/CK19 (Abcam ab52625, 1:200), EPCAM (Abcam ab71916, 1:200), CD133 (Miltenyi Biotec), CD34 (Abcam ab81289, 1:200), KDR/Flk1 (Santa Cruz sc-6251, 1:200), HA (Millipore 05-904, 1:400), DLX5 (Santa Cruz sc18151, 1:200), LGR5 (Santa Cruz sc-68580, 1:200), CD184/CXCR4 (conjugate to PE, BD Pharmingen 561734, 1:200), TRA-1-80 (conjugated to Alexa 488, Stemgent 09-0069, 1:200), FOXA2 (Santa Cruz sc-271104, 1:200), CD31 (Cell Signaling 3528S, 1:200), pan-Haemoglobin (Santa Cruz sc-22718, 1:200), SIX6 (Santa Cruz sc-25070, 1:200), OCT4/POU5F1 (R&D MAB1759, 1:200), CK7 (Santa Cruz sc-53263, 1:200), TAL1 (Santa Cruz sc-12984, 1:200), AQP1 (Santa Cruz sc-32737, 1:200), HNF4α (Cell Signaling Technology 3113S, 1:200), PDGFRα (Cell Signaling Technology 5241P, 1:200), AFP (Santa Cruz sc-8399, 1:200), ALB (Bethyl E80-129, 1:200), Gata6 (Abcam ab22600, 1:200), Brachyury (Abcam ab20680, 1:200), AP2α (Abcam ab11828, 1:200), CD43 (Abcam ab89691, 1:200), CD93 (eBioscience, 14-0939-80), PAX6 (Abcam ab78545, 1:200), CD51 (Abcam ab16821, 1:200) and CD146 (Santa Cruz sc-18837, 1:200). Secondary antibodies used were donkey Alexa Fluor 488 and 597 or 488, 546 and 633 conjugates, respectively (Invitrogen, 1:500). Epifluorescence images were acquired using a Zeiss Axiovert 200M microscope equipped with a 1,344 × 1,024 pixel cooled ORCA-ER CCD camera (Hamamatsu Corporation) and a × 10 objective. Fluorescence images were analysed with the Axiovision digital image-processing package (Zeiss). Confocal images were taken using a Leica TCS SP5 II 405UV confocal microscope (Leica Microsystems, Bannockburn, IL) or Nikon A1R Ultra-Fast Spectral Scanning Confocal Microscope. Images were acquired using a sequential scan for the respective fluorophores.

### Flow cytometry staining

For surface staining, hiPSCs were collected with Accutase, washed and resuspended with conjugated antibodies diluted in Hank's Balanced Salt Solution (HBSS) without Ca^++^ and Mg^++^ with 2% fetal bovine serum for 15 min at 4 °C. Dead cells were labelled using 7-AAD (1:40; BD Biosciences). The cells were then washed and analysed using LSR-II machine or BD Accuri C6 flow cytometer. For intracellular HNF4α staining, cells were collected with Accutase, washed, fixed and permeablized for intracellular staining using the Foxp3 intracellular staining kit (eBioscience). The cells were then blocked with 5% donkey serum, washed and resuspended in primary HNF4α antibody (diluted 1:400) for 30 min at room temperature. The cells were washed again and incubated for 30 min at room temperature with the appropriate fluorophore-conjugated secondary antibody, washed and analysed by flow cytometry. For counting cell numbers, cultures were also stained with a 1:200 dilution of DRAQ5 Fluorescent Probe (Life Technologies) in HBSS with 2% fetal bovine serum. Total number of cells was deduced from number of stained nuclei in the indicated volume measured on BD Accuri C6 flow cytometer.

### Proliferation assay

hiPSC GATA6-2A-EGFP cells were treated with the CellTrace Violet Cell Proliferation Kit, for flow cytometry (Life Technologies) according to the manufacturer's protocol 1 day after seeding, and immediately induced with 1000, ng ml^−1^ Dox in mTeSR1 cell culture media for 4 days. Cultures were then collected as single-cell suspensions by incubating in Accutase for 7 min in at 37 °C, spundown at 300*g* for 5 min, resuspended in HBSS with 2% fetal bovine serum, and kept on ice for flow cytometry analysis on LSR-II machine.

### MethoCult colony-forming unit (c.f.u.) assay

*In vitro* functional assay for enumerating multipotential and lineage commitment of haematopoietic progenitors was performed based on Stemcell Technologies MethoCult c.f.u. assay. CD34^+^ cells were isolated on day 14 using Miltenyi Biotech's MACS technology, washed in IMDM media, and added to MethoCult medium (Stemcell Technologies) in multiple 35 × 10 mm plates at 50, 200 and 500 k cells per plate, and were incubated for an additional 14 days. The number and type of developed colonies were then assessed using an Olympus CK2 inverted microscope on day 14 based on Stemcell Technologies guidelines for colony identification.

### ELISA assays

Collected media were assayed for AAT (Genway Biotech), fibrinogen (Genway Biotech) Oncostatin M (Abcam) and albumin (Bethyl Labs) using commercially available ELISA kits.

### Magnetic isolation of cells

CXCR4 and CD34 isolations were performed at days 5 and 10, respectively, using mouse anti-CXCR4 and mouse anti-CD34 antibodies conjugated to magnetic beads (MACS beads, Miltenyi Biotech). The suspensions were then flowed through Miltenyi magnetic bead LS separation columns alongside a magnet to trap the positive cell population. The columns were then removed from the magnets and flushed out with MACS magnetic bead isolation buffer. A small sample was taken for flow cytometry analysis. Fluorophoreconjugated CXCR4 and CD34 antibodies targeting different epitopes than the bead-conjugated antibodies were used for flow cytometry analysis. The rest of the isolated cells were used for RNA extraction.

### RNA extraction and gene arrays

Total RNA was extracted from cell pellets using the Arcturus Picopure Kit (Ambion/Life Technologies), stored at −80 °C and submitted to the MIT BioMicro Center (Cambridge, MA) where quality control, processing and data acquisition for the microarray (SurePrint G3 Human Gene Expression 8x60K v2 Microarray Kit, Agilent) was performed according to the manufacturer's instructions (Single Color Hybridizations). Gene Array data was analysed using Genespring GX 12 (Agilent) in the Standard Guided Workflow (Technology: Agilent.SingleColor.28004, normalization: shift to 0.75 percentile, baseline transformation: median of all samples) with triplicates for each condition. Heatmaps were generated based on normalized gene array data using R and the heatmap.2 package (www.r-project.org/).

### Gene set enrichment analysis

Genes >4x differentially upregulated between days 5 and 10 (*N*=403) or days 10 and 15 (*N*=309) in total cells RNA extractions were uploaded to the Broad Institute Gene Set Enrichment Analysis analysis platform (www.broadinstitute.org/gsea/) and queried against the Biocarta and KEGG gene sets with an FDR (estimated false discovery rate) value smaller than 0.05.

### Cell sorting by flow cytometry

Gata6-2A-EGFP engineered hiPSCs were induced for 2 days, detached from the plates with Accutase (Stemcell Technologies), and suspended at 2 million cells per ml in mTeSR-1 supplemented with 10 μM of Y-27632 and 0.6 μM Thiazovivin (Tocris Biosciences). The high GATA6-expressing cells were separated by EGFP fluorescence signal using a BD Aria III cell sorter. The separated population was reseeded into BD 24-well plates on matrigel-coated glass coverslips in mTeSR containing 1,000 ng ml^−1^ Dox and switched to APEL at day 4. The cultures were monitored for 9 days in APEL media.

### Ectodermal outgrowth isolation

Ectodermal outgrowths were identified visually using a EVOS microscope (Life Technologies) and picked with a glass capillary connected to a mouth-operated vacuum. Three times 35 outgrowths were picked and immediately subjected to RNA extraction using the Arcturus Picopure Kit (Ambion/Life Technologies).

### Pharmacological study (Axitinib)

hiPSCs were differentiated as described in our cell differentiation protocol. Non-pluripotency supporting medium (APEL, Stem Cell Technologies, Vancouver), was then supplemented with Axitinib to a final concentration of 50 nM. An equal volume of DMSO was used as control. Cells were then fixed, stained and imaged, as described in our immunofluorescence protocol.

### Time lapse Imaging

Gata6 engineered iPS cells were seeded at 26 K cells per cm^2^ in a BD six-well tissue culture treated plate coated with BD Matrigel, then induced with 1,000 ng ml^−1^ Dox. After induction, the cells were viewed under a Leica DMI6000 Confocal Laser Scanning Microscope, in an incubation chamber. Images were taken every 30 min for 4 days.

### Image analysis (Cellprofiler)

Images were analysed and quantified using Cellprofiler 2.1.0 (www.cellprofiler.org) with a custom pipeline, sequentially applying the following modules to each image channel: ApplyThreshold, IdentifyPrimaryObjects, IdentifySecondaryObjects, RelateObjects, MeasureObjectIntesity and ExportToSpreadsheet. To investigate area covered by CD34^+^ and AAT^+^ cells, representative area of 1.02 mm^2^ or greater was used in at least three independent samples and analysed using the Identify Primary Objects module to measure the percentage of total tissue area for which AAT or CD34 stain is detected.

### Statistical analysis

ELISA data are expressed as the means +/− s.e. of the mean (s.e.m.) from three independent measurements. Gene array normalization was performed using percentile Shift and downstream quality control was done on biological triplicates using the Genespring Standard Guided Workflow (Agilent).

## Additional information

**Accession codes:** All microarray data have been deposited in the Gene Expression Omnibus database under accession code GSE74662.

**How to cite this article:** Guye, P. *et al*. Genetically engineering self-organization of human pluripotent stem cells into a liver bud-like tissue using Gata6. *Nat. Commun.* 7:10243 doi: 10.1038/ncomms10243 (2016).

## Supplementary Material

Supplementary Information Supplementary Figures 1-21, Supplementary Tables 1-3, Supplementary Note 1 and Supplementary References

Supplementary Movie 1 4 days time lapse movie from day 1 to 5. Cells were seeded in medium containing Dox and timelapse imaging was initiated the next day with images taken every 30 minutes for 90 hours (~4 days). The movie shows self-sorting of the cells with formation of clear boundaries between differentiated cells and pluripotent cells.

Supplementary Movie 2 A 3D rotational movie of the organoid on day 18. Tissue was fixed and stained for AAT (red) and CD34 (green) with Hoechst (blue). Images were captured and rendered from a Nikon A1R confocal microscope, and show the presence of cell layers in culture.
