# Supplementary material for: Genetically engineering self-organization of human pluripotent stem cells into a liver bud-like tissue using Gata6
Source: Nat Commun. 2016 Jan 6;7:10243. doi: 10.1038/ncomms10243 (PMC4729822; doi:10.1038/ncomms10243)
Supplement: Supplementary Information — Supplementary Figures 1-21, Supplementary Tables 1-3, Supplementary Note 1 and Supplementary References [file ncomms10243-s1.pdf]

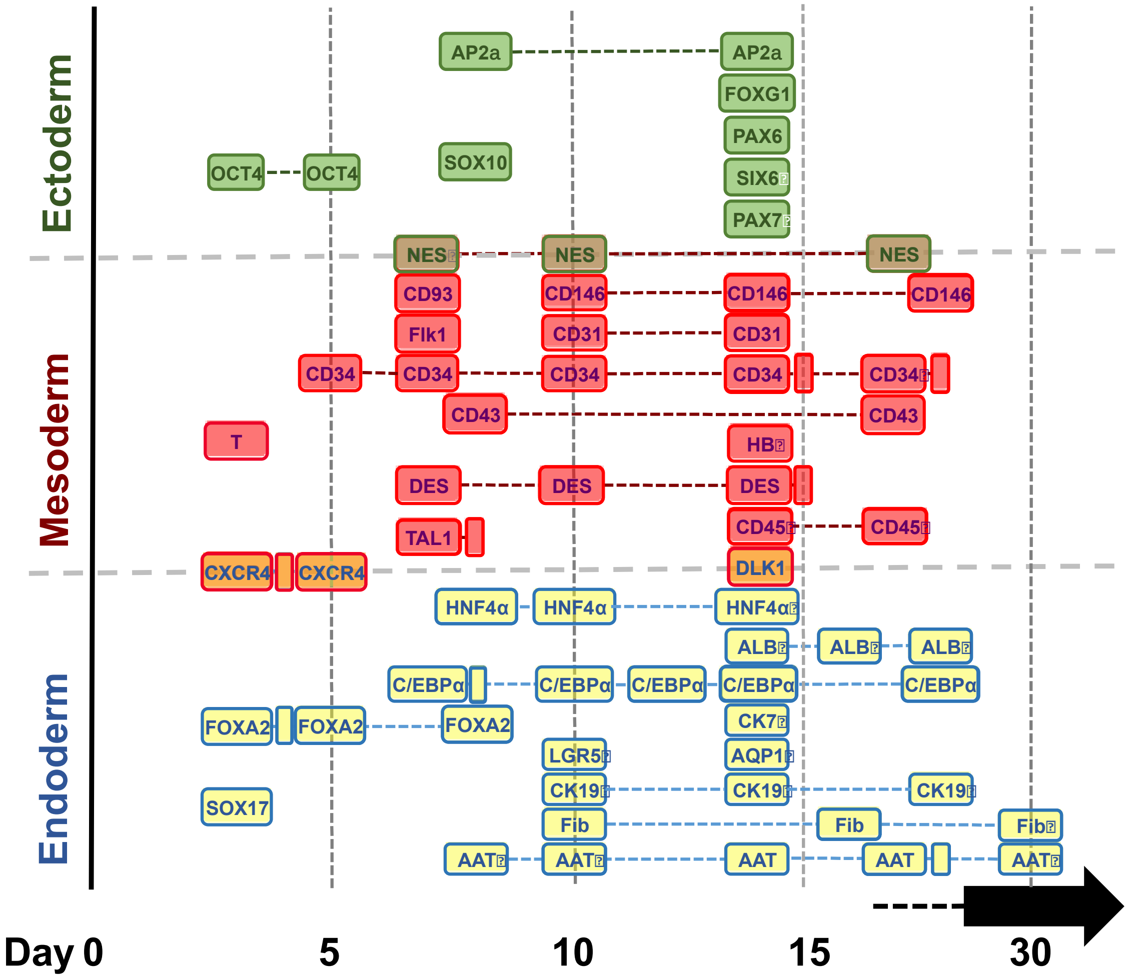

Supplementary Figure 1 | Temporal expression of key cellular markers observed during the development of our organoid. Endoderm markers (FOXA2, SOX17) and mesoderm (T) are observed around day 3 (Fig. 2f; Supplementary Fig. 2 and 3). CXCR4+ cells develop and expand after day 3 (Fig. 3a,b). The endoderm matures further towards a hepatic fate between days 5 and 10 while in parallel CD34$^+$ cells emerge on day 6 (Fig. 4). CD34$^+$ cells acquire CD146 and CD31, connect to form endothelial tube-like structures by Day 14 (Fig. 4b, 5a, 5c). Some nestin$^+$ (NES) cells appear on Day 7 and some reside in a close proximity to the vascular-like structures, resembling microvascular pericytes on day 17 (Fig. 4c, 6f, Supplementary Fig. 9, 10). Desmin$^+$(DES) DLK1$^+$ cells with stellate cell morphology appear around day 10 (Fig. 5b, Supplementary Fig. 8a). Most fetal hepatocyte-like cells express the markers fibrinogen (Fib), AAT, CEBPα, LGR5, HNF4α, CK19 and DLK1 between days 10 to 14 (Fig. 5, Supplementary Fig. 7, 20). Albumin (ALB) protein also increases between days 14 to 18 (Fig. 5f). Cholangiocyte-like cells (CK7$^+$ and AQP1$^+$) are observed within the fetal hepatocyte-like cell layer around day 14 (Fig 5e, Supplementary Fig.11). A population of CD34+ cells on day 7 express CD93, TAL1 and Flk1 resembling hemangioblast cell phenotype (Fig 4a, 6e Supplementary Fig. 7c, d). They express CD43$^+$ on day 8 suggesting a hematopoietic specification (Fig. 6e, Supplementary Fig. 10a). On Day 14 and 17, small spherical cells expressing CD45 or hemoglobin are detected inside and around CD34$^+$ endothelial tubes

suggesting hematopoiesis-like processes (Fig. 6b,c, Supplementary Fig. 9 and 10b). In distinct clusters of non-transduced cells, the pluripotency and ectoderm marker OCT4 increases in the first five days, and they subsequently acquire ectodermal markers ($AP2a^{+}$, $SOX10^{+}$) on day 10, neural crest and neuronal markers (FOXG1, PAX6, SIX6, PAX7) on day 14 (Supplementary Fig. 17). Boxes represent the time points at which markers were observed. Association of each marker with germ layers was selected based on available literature and depicted by a different color. Combined colors show a relation to more than one germ layer.

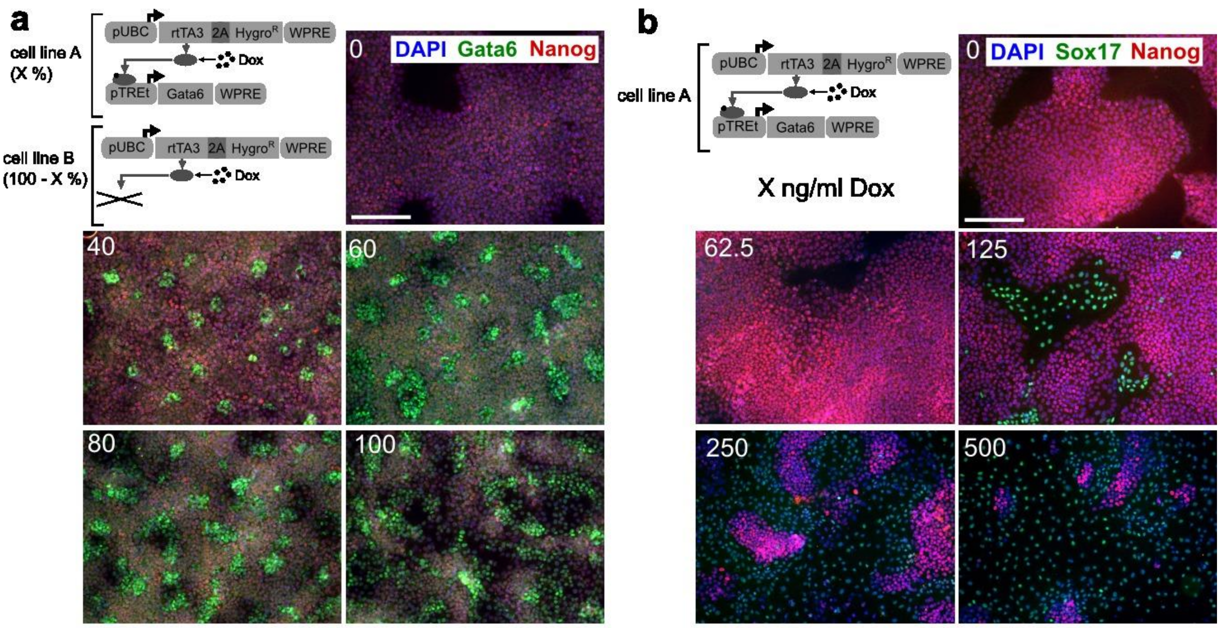

Supplementary Figure 2 | Spatial segregation of Gata6$^+$ and Nanog$^+$ populations. (a) Mixing GATA6-expressing and non-expressing cells enables modulation of the endoderm to non-endoderm ratio within the population (day 3). Scale bar: 200 µm. **(b)** Dox-titration curve and impact on endoderm generation. Higher Dox concentrations increase the ratio of endoderm to non-endoderm cells within the population (day 3). Scale bar: 200 µm.

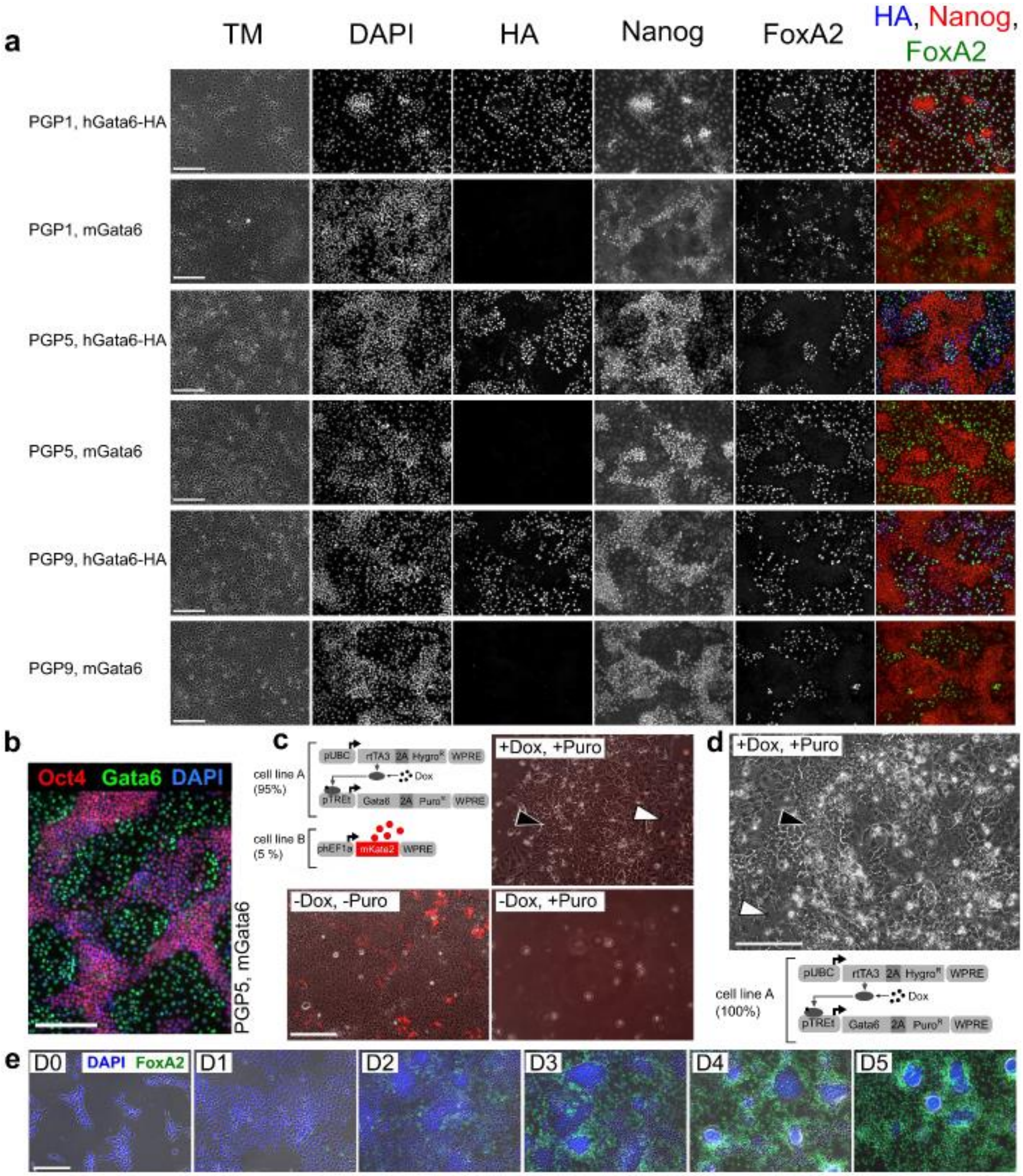

Supplementary Figure 3 | Segregation into GATA6^{+} endodermal cells and clusters of Oct4^{+} and Nanog^{+} cells. (a) Expression of ectopic GATA6 and segregation into Nanog^{+} and FoxA2^{+} sub-populations on day 3. m: murine, h: human Gata6. **(b)** Segregation into Oct4^{+} and GATA6^{+} sub-populations. **(c, d)** Puromycin kills uninduced and WT (cell line B) cells. Black arrow: compact, pluripotent-like cluster, White arrow: flat endodermal-like cell. **(e)** FoxA2 daily immunostaining. Dx=Day x. Scale bar: 200 μm.

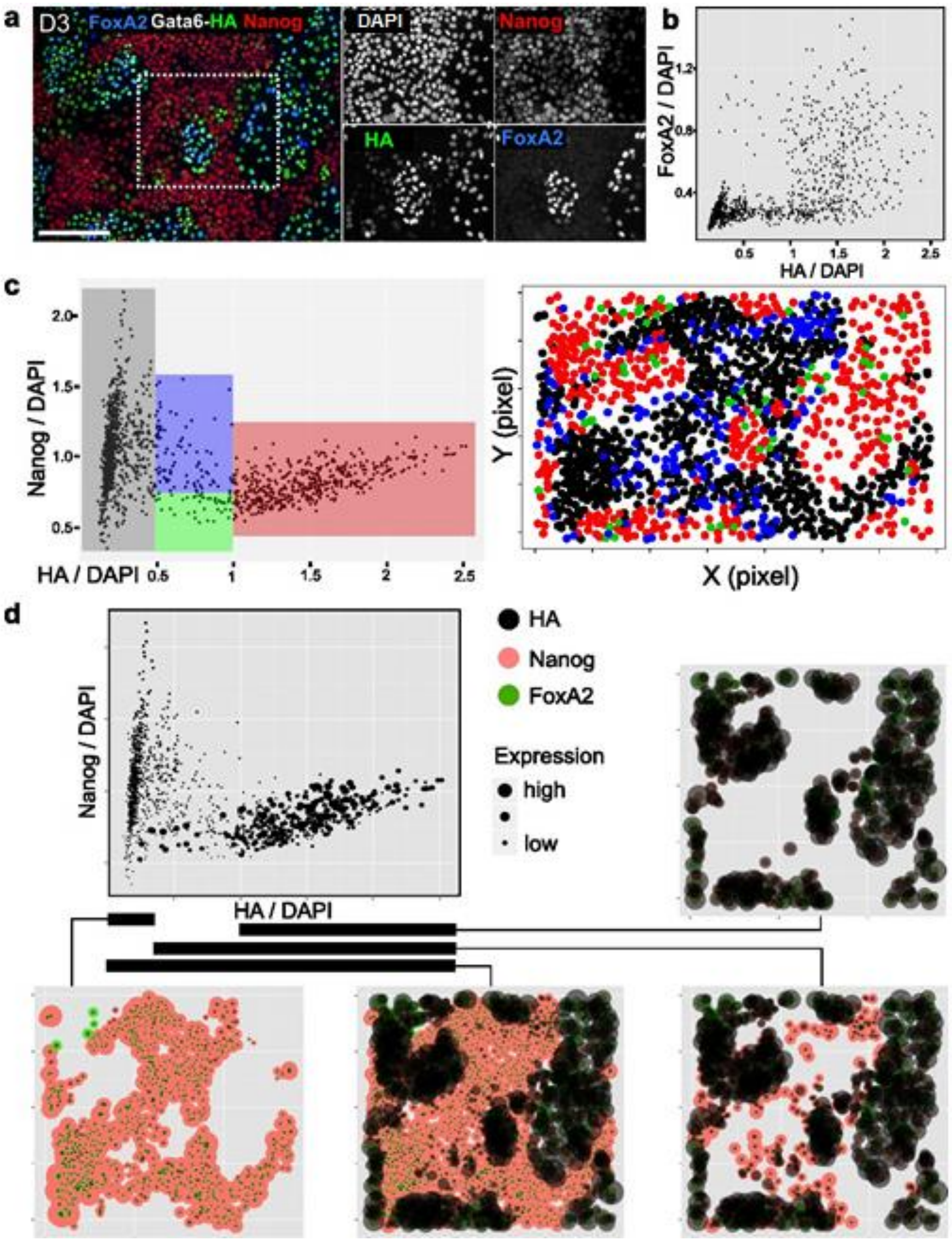

Supplementary Figure 4 | Single cell analysis of symmetry breaking. (a) Original image (Fig 2g) with an enlarged section for visualizing the separate channels. **(b)** GATA6 expression, as detected by HA staining, must reach a defined threshold for FoxA2 to be expressed. **(c)** Cells with low GATA6 expression levels localize to the endoderm sub-population if Nanog is low, otherwise they localize to the Nanog$^+$ sub-population. Areas in the scatter plot (left) are mapped to the cell X/Y positions (right) with the respective colors. **(d)** Binning of the scatter plot (Fig. 2h) mapped back to the X/Y cell positions. The size of each event in the left scatter plot is indicative of its FoxA2/DAPI level. Each value is normalized by its corresponding DAPI signal. DAPI: nuclear (DNA) signal. Scale bar: 200 µm.

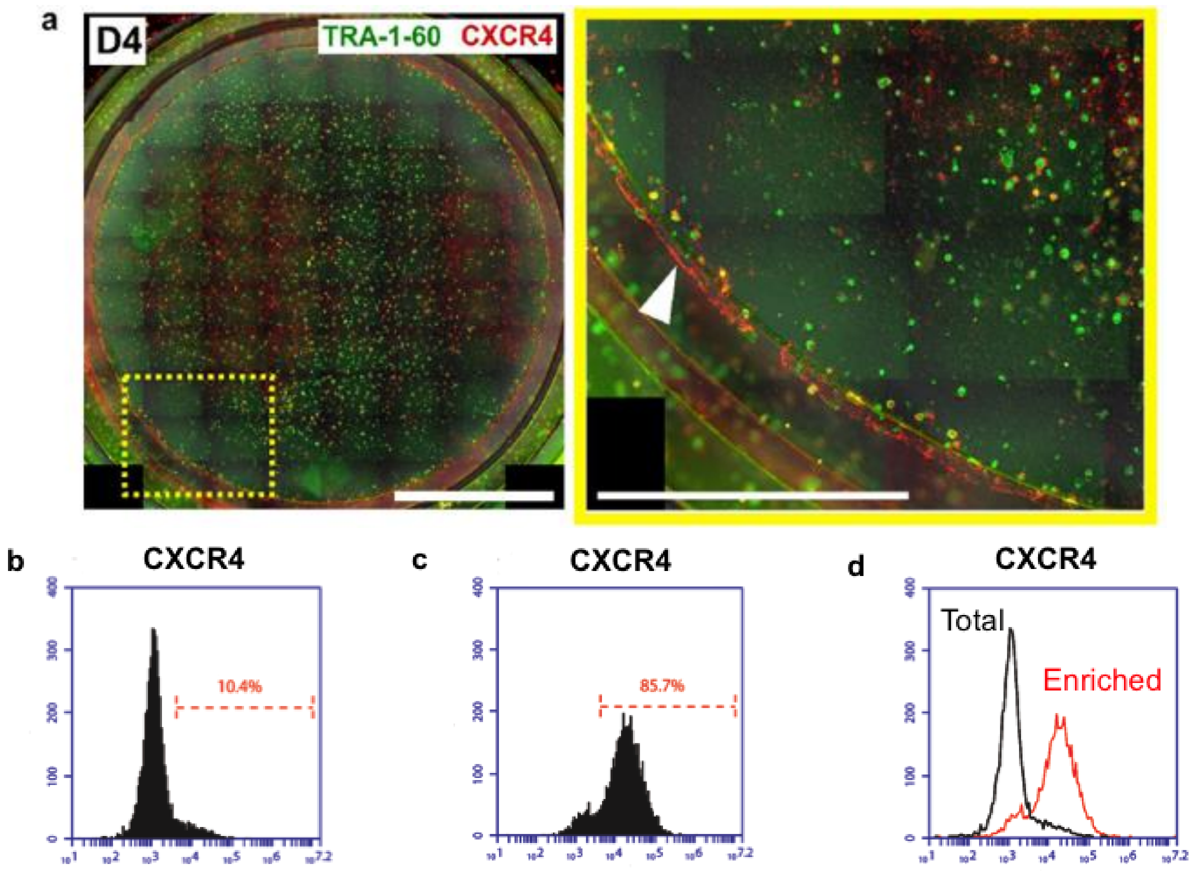

Supplementary Figure 5 | CXCR4 subpopulation and its isolation. (a) GATA6-induced CXCR4$^+$ cells: CXCR4$^+$ cells are hypermotile and localize to the edges of the endodermal layer. Left scale bar is 1 cm and right scale bar is 0.5 cm. **(b-d)** Flow cytometry analysis of CXCR4$^+$ cell isolated **(b)** from the total cell population **(c, d)** shows 8.2 fold enrichment in CXCR4 expressing cells.

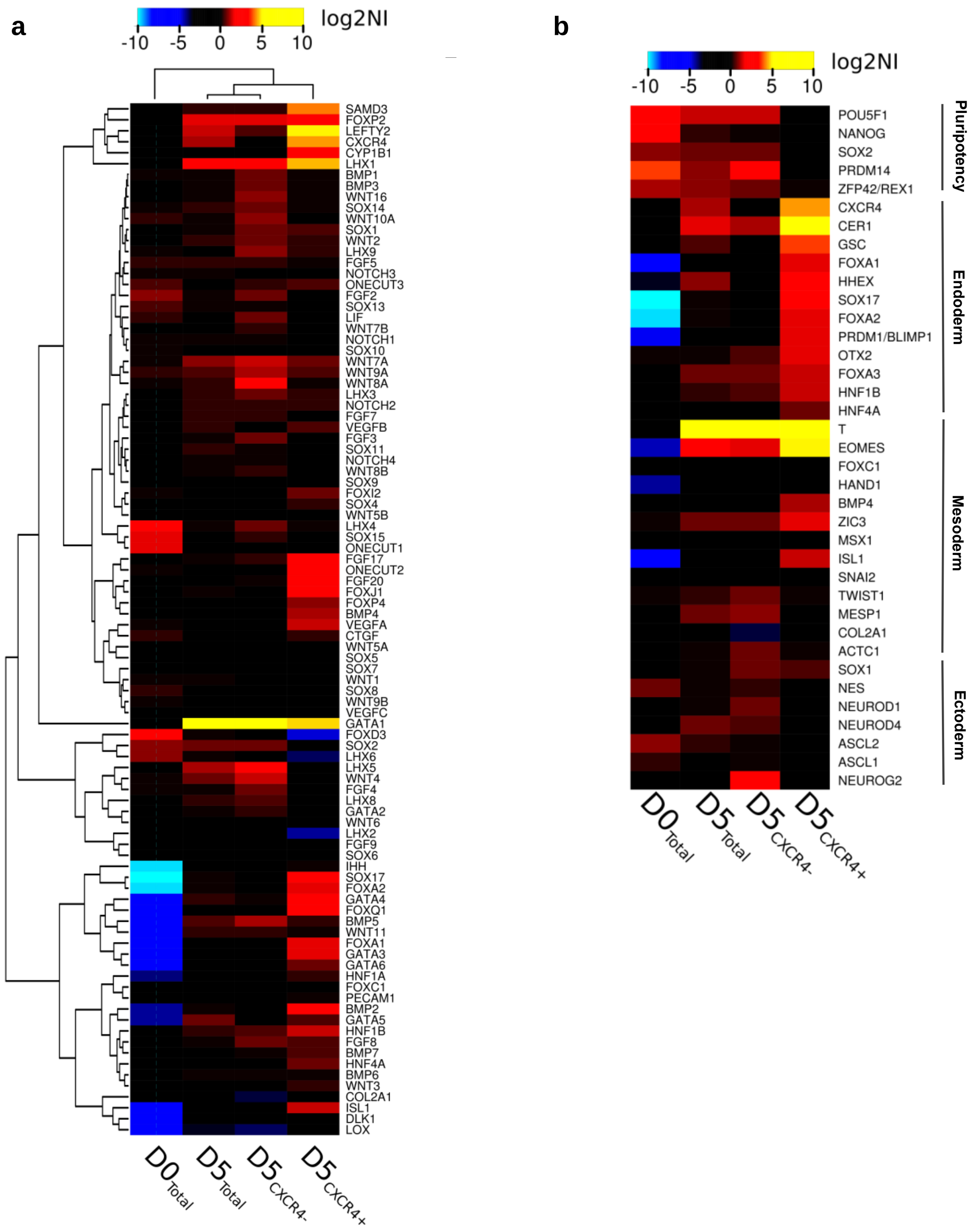

Supplementary Figure 6 | Transcriptional analysis of cells. (a) Comprehensive heatmap view of various genes regulating cell fate determination of human induced pluripotent cells (hiPSCs) in uninduced hiPSCs on day 0, total Gata6-induced cells versus the isolated CXCR4^{+} and CXCR4^{-} cell fractions on day 5. **(b)** Heatmap clustering of gene expression data for markers specific for endoderm, mesoderm and ectoderm.

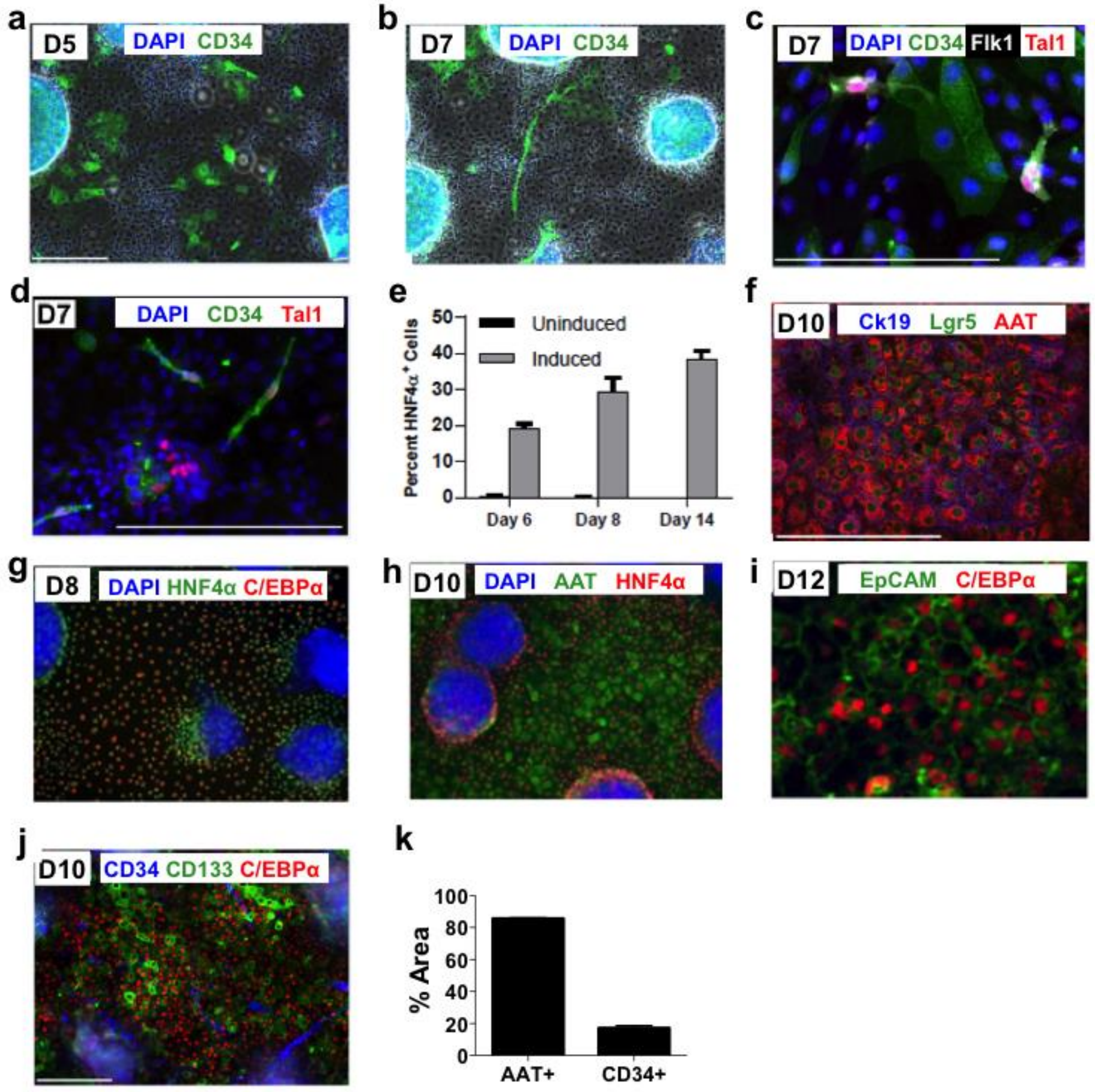

Supplementary Figure 7 | Maturation of specific populations. **(a)** CD34$^+$ endodermal-like cells at day 5 and **(b)** CD34$^+$ endothelial-like cells at day 7. **(c)** TAL1$^+$, CD34$^+$ and flk1$^+$ cells on day 7. **(d)** CD34 and TAL1 show hematopoietic commitment of cells. **(e)** Increasing percentages of HNF4α$^+$ cells to total cells. Data are mean ± S.E.M and representative of 2 cultures per group **(f)** Confocal image of Lgr5 and AAT co-expression in the hepatoblasts at day 10. **(g)** CEBPα$^+$ and HNF4α$^+$ expression on day 8. **(h)** AAT$^+$ and HNF4α$^+$ expression on day 10. **(i)** EpCAM$^+$ and CEBPα$^+$ expression on day 12. **(j)** CD133$^+$ and CEBPα$^+$ expression on day 10. **(k)** Areas covered by AAT$^+$ and CD34$^+$ cells in cultures between Day 14 to 18. Data are mean ± S.E.M. n=3 for AAT$^+$ and n=5 for CD34$^+$ cells. Scale bars: 200 µm.

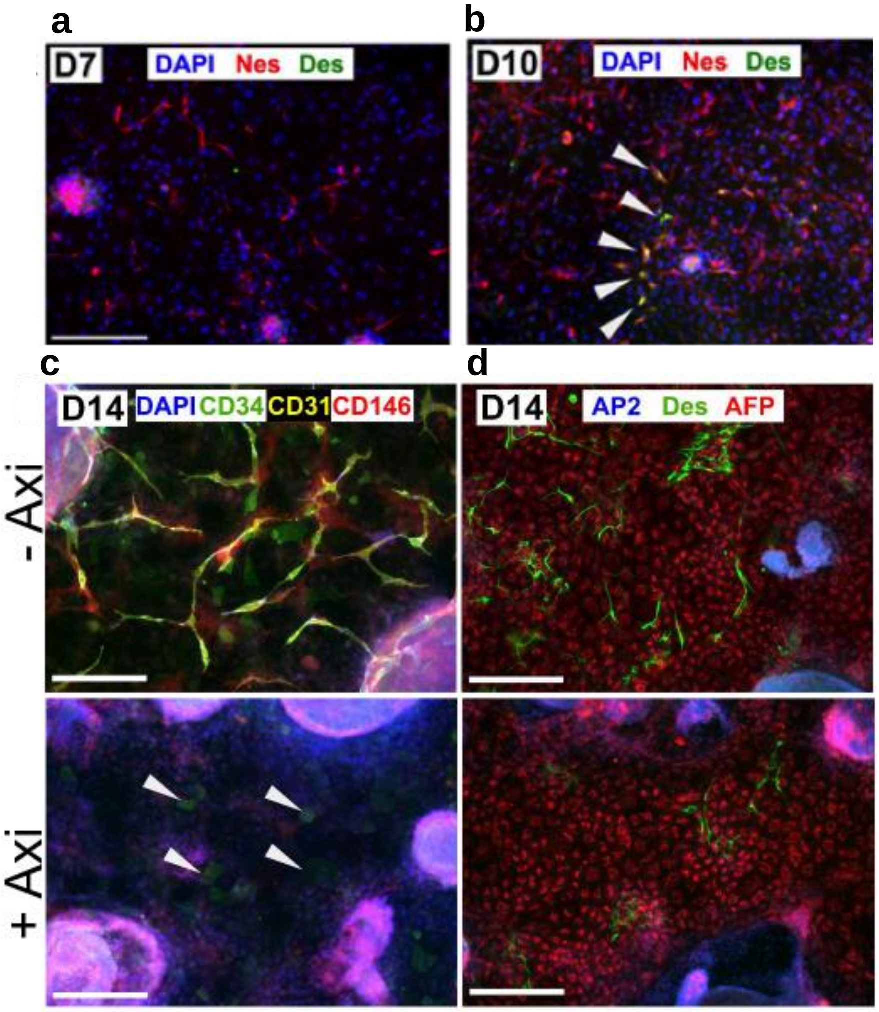

Supplementary Figure 8 | Nestin and desmin expression and Axitinib mediated regulation of angiogenesis. (a) Staining for Nestin (Nes) and Desmin (Des) on day 7 **(b)** Des+ Nes+ cells on day 10. **(c)** Axitinib (Axi) abolishes the emergence of $CD34^+$ endothelial-like cells, but not $CD34^+$ endodermal-like cells (arrow heads). **(d)** A reduced but not abolished number of DES^+ cells are observed in Axitinib-treated cells. AFP (red) marks fetal hepatocyte-like cells and AP2 (blue) marks the ectodermal/neuronal cell clusters. Scale bars: 200 µm.

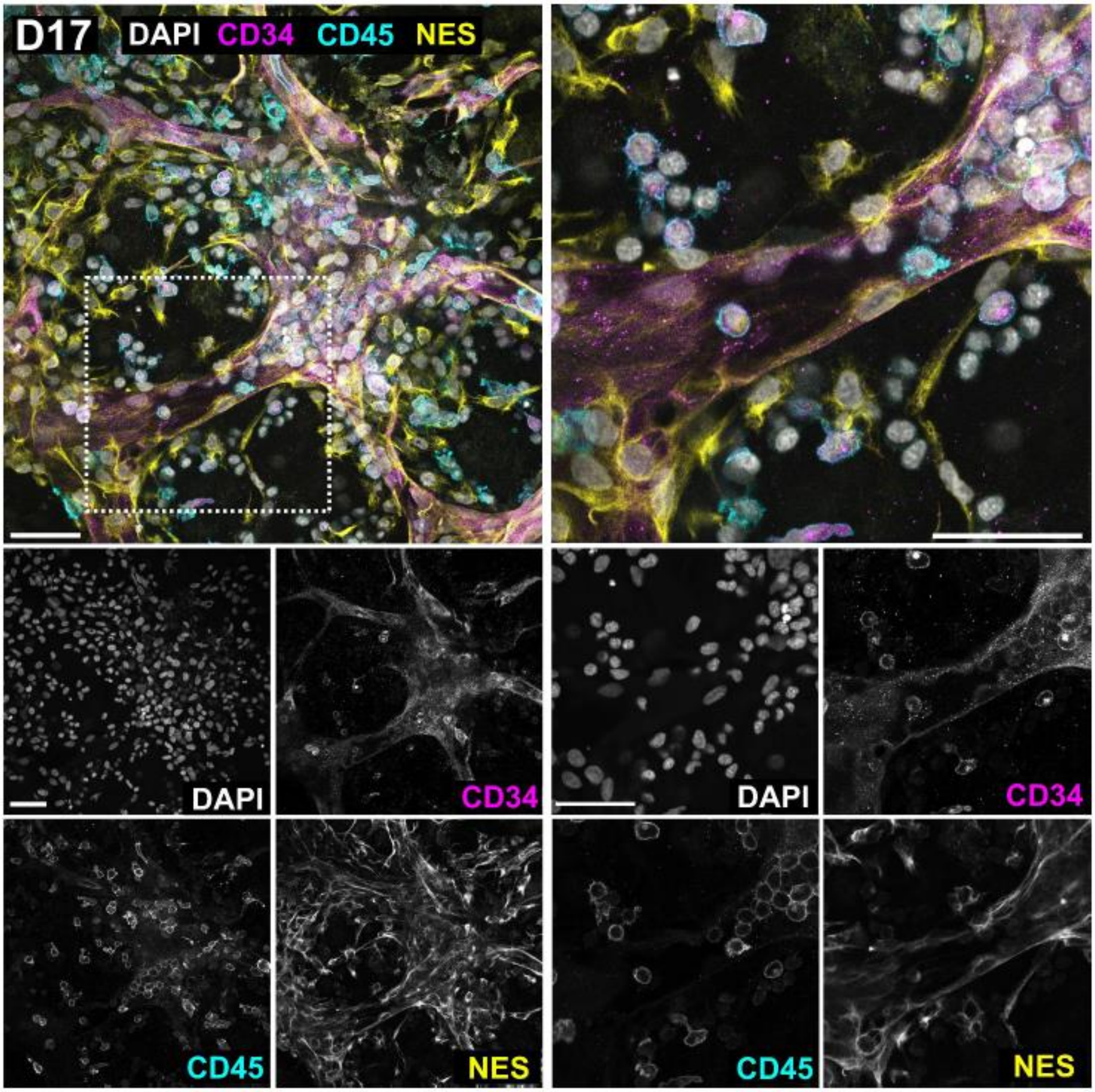

Supplementary Figure 9 | Mesenchymal and pericyte like cells in proximity to the endothelial tubes and haematopoietic progenitors. Confocal images were acquired through sequential scans with the respective laser lines (right panel: enlarged section of endothelial tube-like structure). $CD34^{+}$ tube-like structures covered in pericyte-like cells (NES^{+}) acts as centers of emerging haematopoietic-like cells ($CD45^{+}$). Sale bars: 50 µm. D17: Day 17.

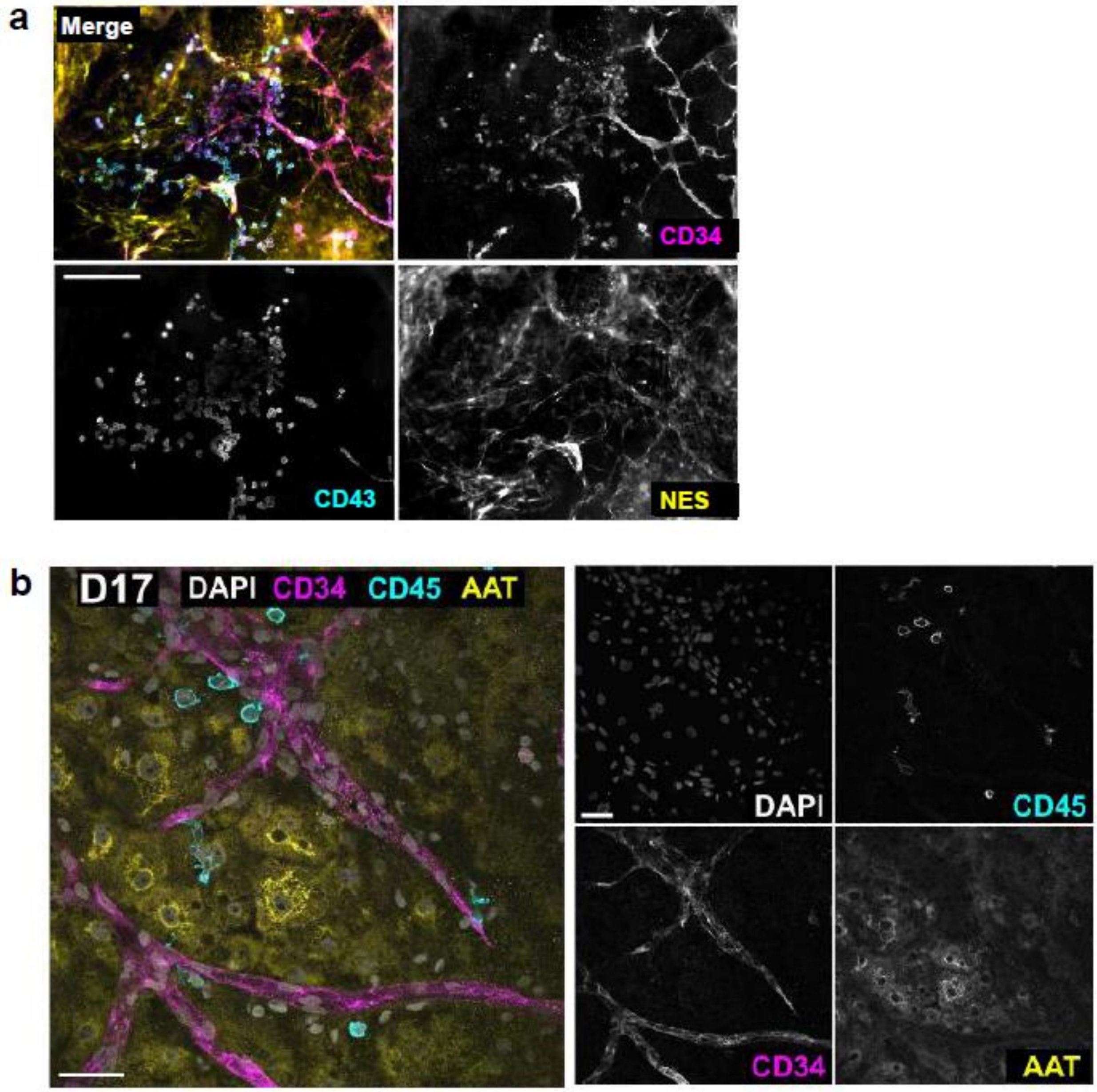

Supplementary Figure 10 | Codevelopment of different subset of cells. (a) CD34$^+$ endothelial tube-like structures covered in pericyte like cells (NES$^+$) that contain developing haematopoietic progenitor-like cells (CD34$^+$ CD43$^+$) on day 17. Scale bar: 200 µm. **(b)** CD34$^+$ endothelial tube-like structures with developing haematopoietic-like cells (CD45$^+$) embedded within fetal hepatocyte-like cells (AAT$^+$). Sale bars: 50 µm. D17: Day 17. Confocal images were acquired through sequential scans with the respective laser lines.

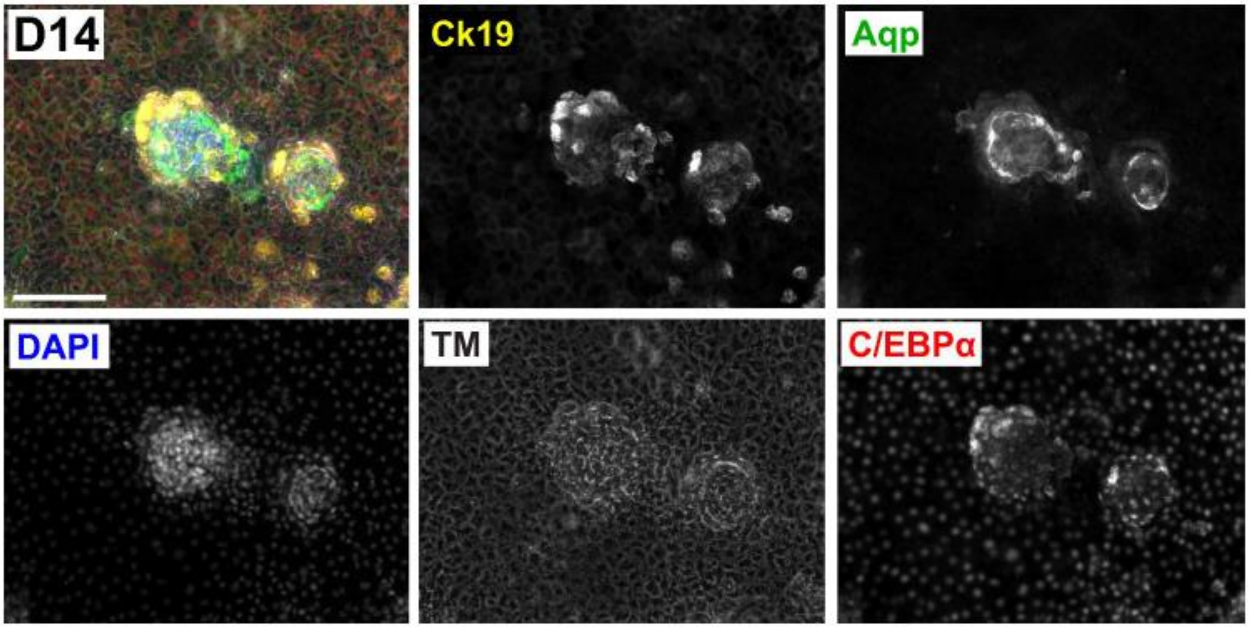

Supplementary Figure 11 | Cholangiocyte like cells develop within the hepatocyte like layer. Immunostaining on Day 14 for Ck19 and Aquaporin (Aqp) shows cholangiocyte-like cells. Scale bar: 200 μm.

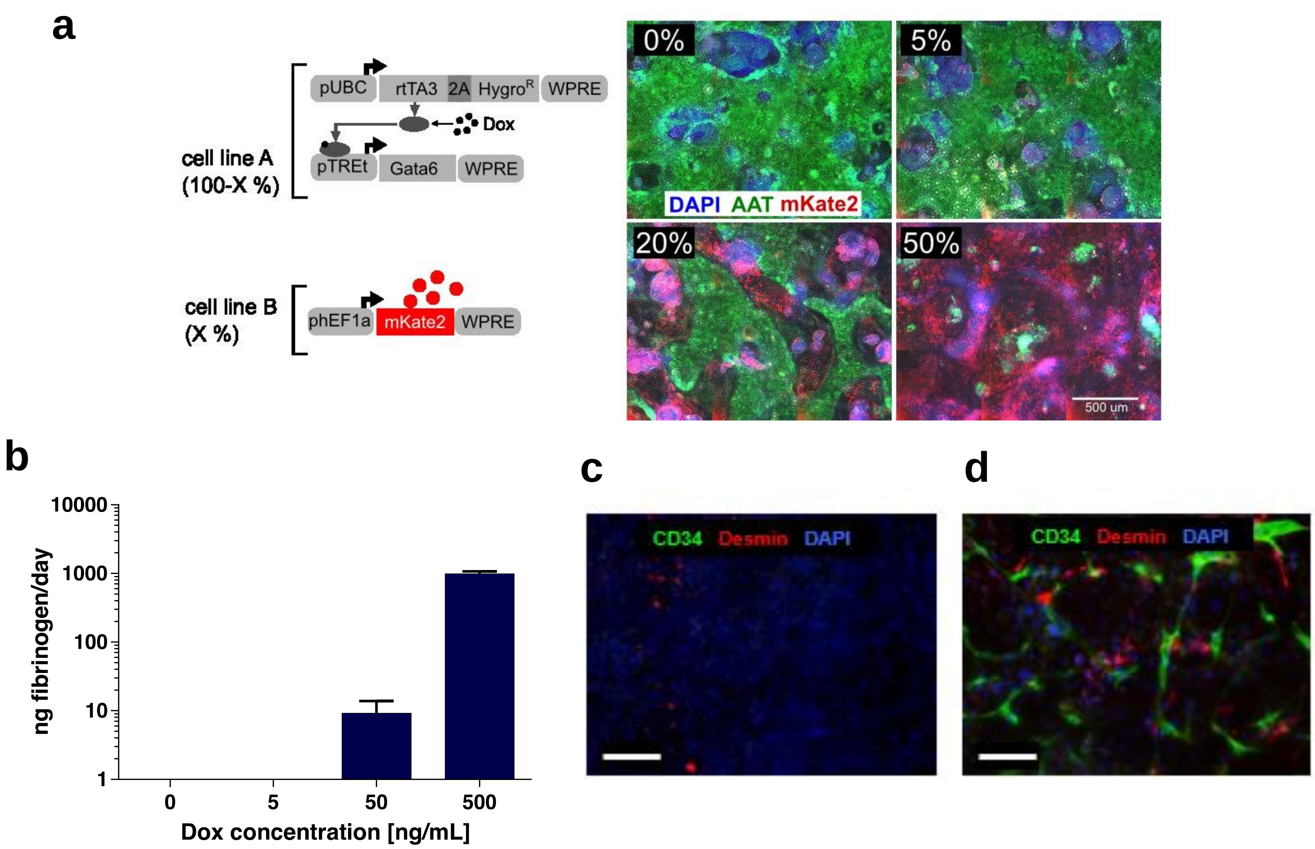

Supplementary Figure 12 | Modulation of endoderm and mesoderm development by altering the ratio of Gata6$^+$ cells or the level of Gata6. (a) Mixing GATA6-expressing and non-expressing cells enables modulation of the portions of the cells within the population that are fetal hepatocyte-like cells (AAT staining, day 14). Scale bar: 500 µm. **(b)** Fibrinogen production in tissue on day 14 shows that hepatocyte-like cells development is correlated with Dox dosage and suggests dependence of fibrinogen producing hepatocyte-like cells on expression of GATA6. Data are mean ± S.E.M and representative of at least three cultures per group. Additional staining for mesoderm markers CD34 and desmin on day 15 in **(c)** uninduced cultures and **(d)** cultures dosed with 1000 ng/mL Dox show the presence of very few desmin$^+$ cells, and no CD34$^+$ cells in the uninduced culture, while both markers are developed clearly in the dosed cultures. Scale bars: 200 µm. This suggests dependence of mesoderm development on GATA6 expression.

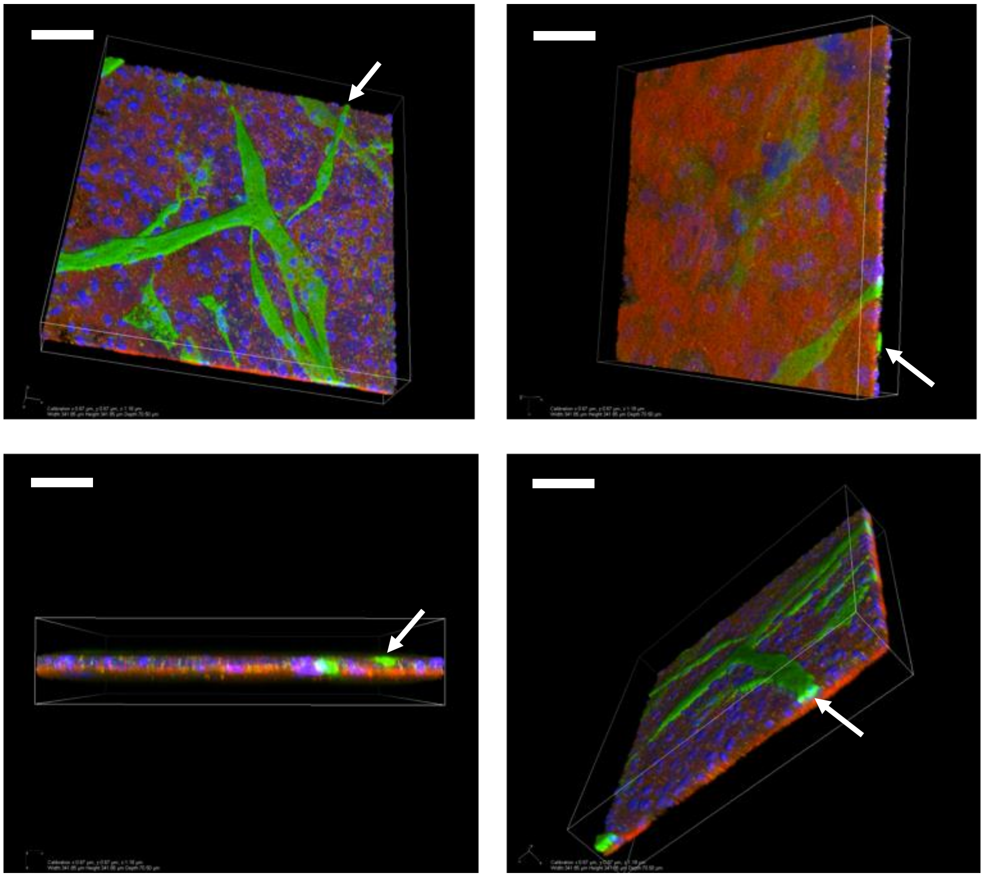

Supplementary Figure 13 | Confocal microscopy of organoid on day 18. Confocal Microscopy shows the tissue develops with multiple layers of cells. The representative images show $CD34^+$ endothelial-like cell tubes (green; CD34 staining) that developed on top of hepatocyte-like cells, marked red for expression of AAT. White arrows show tube-like features usually on top of the hepatocyte layer. Scale bars: 50 µm

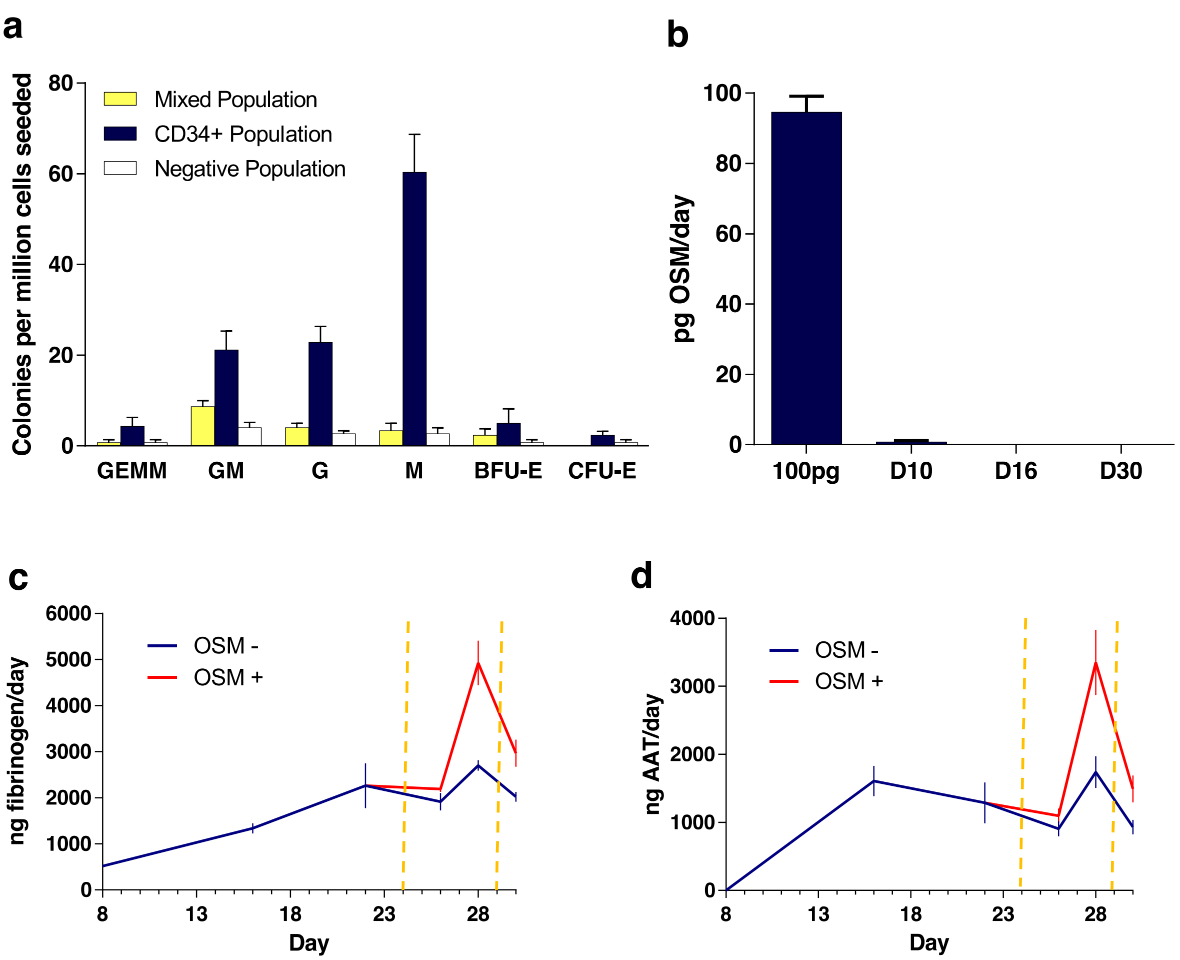

Supplementary Figure 14 | Colony forming unit assay and Oncostatin M treatment. (a) Methocult colony forming unit (CFU) assay shows multipotent potential of $CD34^+$ cell population isolated on day 14. **(b)** Oncostatin M (OSM) measurement in media collected from differentiated cells at multiple time points. **(c, d)** On day 24, half of the cultures were treated with 20ng/mL OSM. Conditioned samples collected from media changes were analyzed for fibrinogen, and AAT, and a significant increase was detected in both proteins after three days of OSM treatment. The yellow dashed lines show the start and end of OSM treatment. Data are mean ± S.E.M and representative of at least three cultures per group.

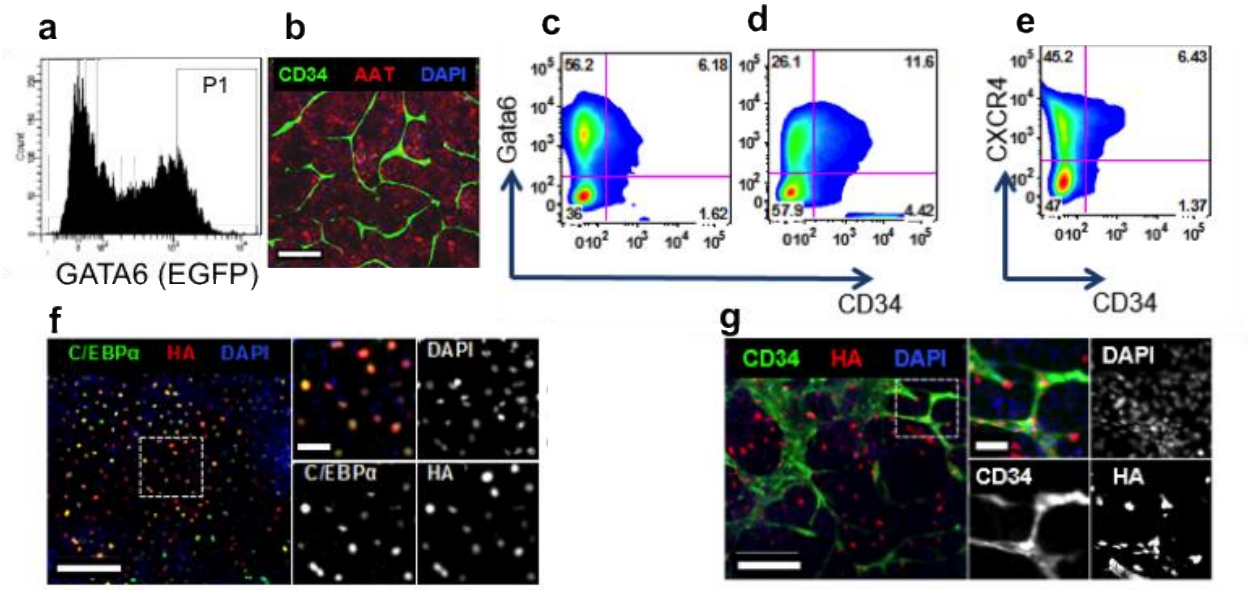

Supplementary Figure 15 | Emergence of CD34$^+$ cells in GATA6 expressing population. (a) *GATA6-2A-EGFP* transduced cells sorted and seeded based on high EGFP fluorescence intensity (P1 gate). **(b)** The tissue at day 14 shows well developed CD34$^+$ vascular-like structures among AAT$^+$ hepatocyte-like cells. Scale bar 200 µm. **(c)** Flow cytometry analysis of differentiated *GATA6-2A-EGFP* transduced cells at day 5 and **(d)** 8 shows a population of CD34$^+$ cells that arises from GATA6$^+$ cells. **(e)** Flow cytometry analysis of differentiating cultures at day 5 also shows a population of CD34$^+$ cells that initiates from CXCR4$^+$ cells. **(f)** Immunostaining for HA in tissue developed from *GATA6-HA* transduced hiPSCs that were redosed with Dox on day 17. The data indicate presence of active *GATA6-HA* transgene in most of C/EBPα$^+$ hepatocyte-like cells and **(g)** in subpopulations of CD34$^+$ endothelial-like cells. Large scale bar: 200µm. Zoomed selection scale bar: 50 µm.

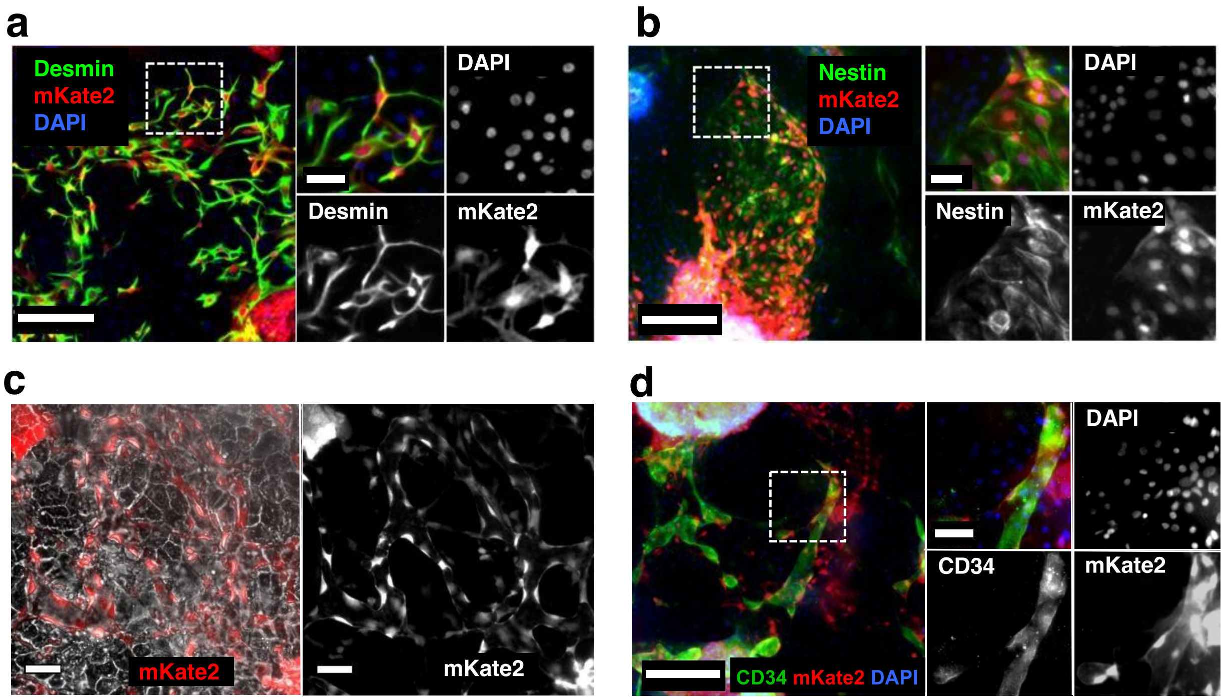

Supplementary Figure 16 | Fate of WT cells in GATA6 expressing population. GATA6 engineered hiPSC and WT hiPSC (constitutively express mKate2) are combined (1:4 ratio), seeded and induced by Dox. **(a)** Overlap of mKate2 and desmin-expressing cells and **(b)** mKate2 and NES-expressing cells indicates that wild type cells can contribute to both DES^+ and NES^+ cell populations by day 20. **(c)** mKate2 expressing WT cells can also contribute to formation of tube-like structures on day 20 and **(d)** $CD34^+$ cell fate. Large scale bars: 200 µm. Zoomed selection scale bars: 50 µm

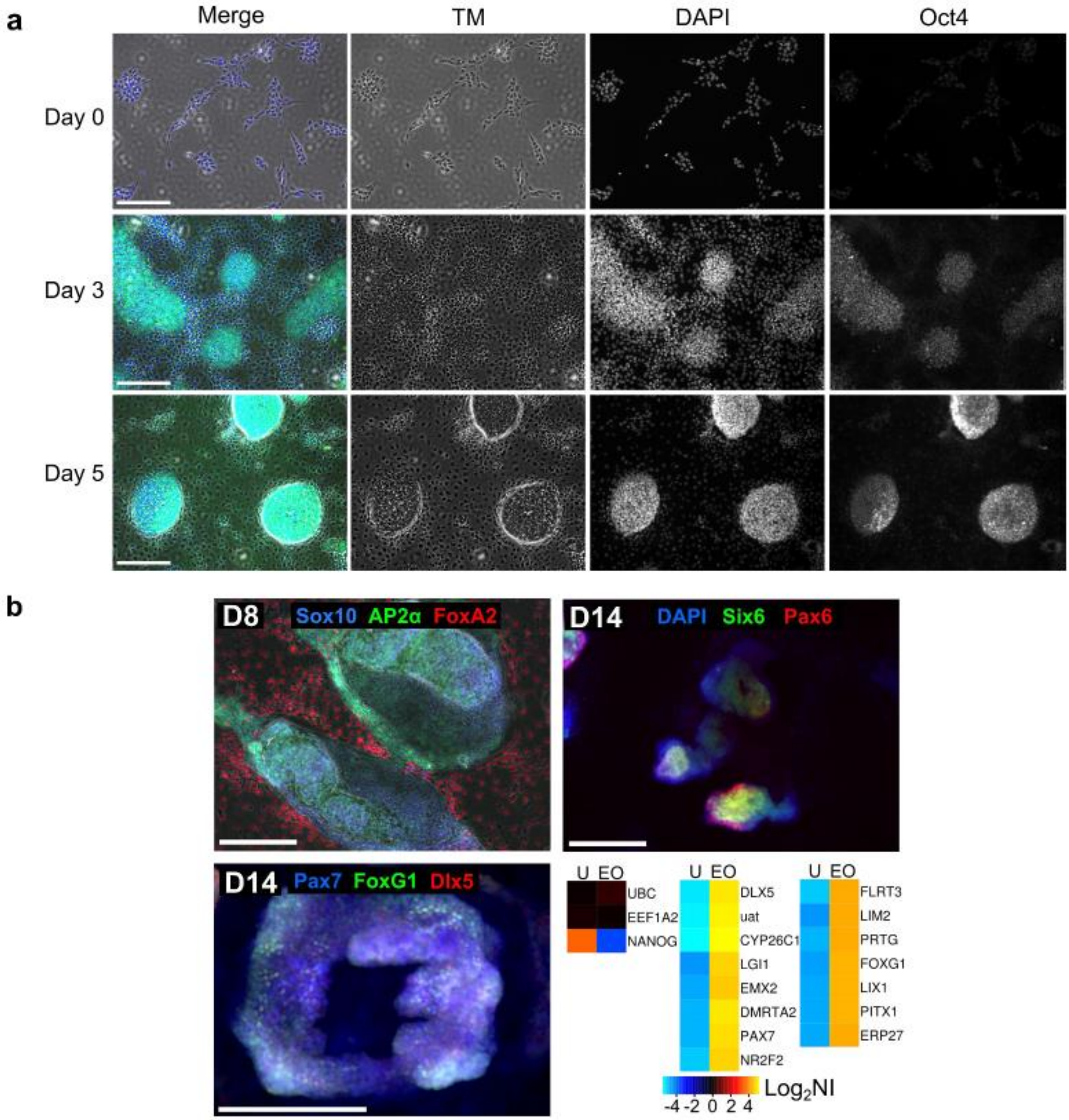

Supplementary Figure 17 | Clusters of cells that have not differentiated to mesendoderm acquire a neural fate. (a) Increased expression of OCT4 in the non-mesendodermal clusters within the first five days. **(b)** Islands of formerly pluripotent cells within the endodermal layer (FOXA2) acquire a neuroectodermal identity ($SOX10^{+}$, $AP2^{+}$). Neuronal progenitors develop separately from the endodermal layer (FOXA2). $Six6^{+}$ and $Pax6^{+}$ cells are observed on D14. $FOXG1^{+}$ and $DLX5^{+}$ cells are observed at the same time point. Heatmap of the 15 most upregulated genes in the neural fold-like tissue and three control genes (UBC, EEF1A, NANOG). U: Uninduced (no Dox), EO: Ectodermal outgrowths. uat = unknown anti-sense transcript. Log_2NI: log_2 normalized intensities. Please refer to the supplementary note 1. Scale bars: 200 µm.

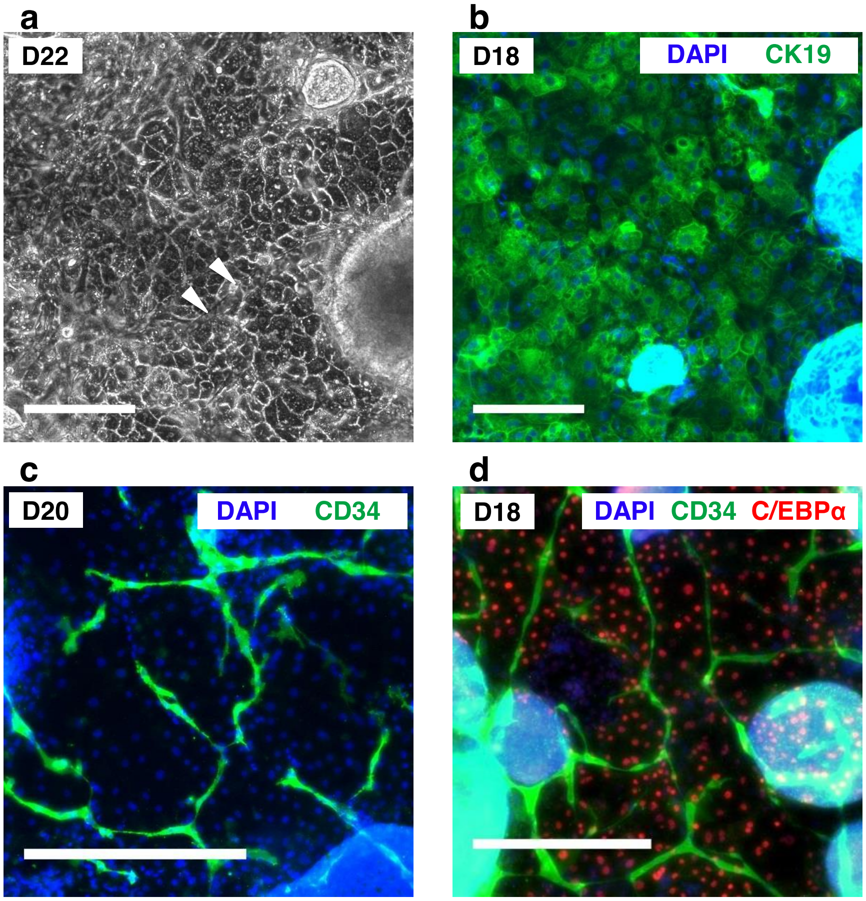

Supplementary Figure 18 | Long-term stability of GATA6 induced human induced pluripotent cell cultures. (a) Brightfield image of cultures on day 22 shows that the hiPSC differentiated cultures maintain distinct cell borders with characteristic polygonal shape of hepatocytes and vessel-like structures (arrow heads). **(b)** Staining of $CK19^{+}$ cells on day 18. **(c)** Staining of $CD34^{+}$ cells on day 20 and double staining for CD34 and CEBPα (**d**) on day 18 show maintenance of vessel network at later time points as well as the presence of CEBPα $^{+}$ hepatocyte-like cell. Scale bars: 200 µm.

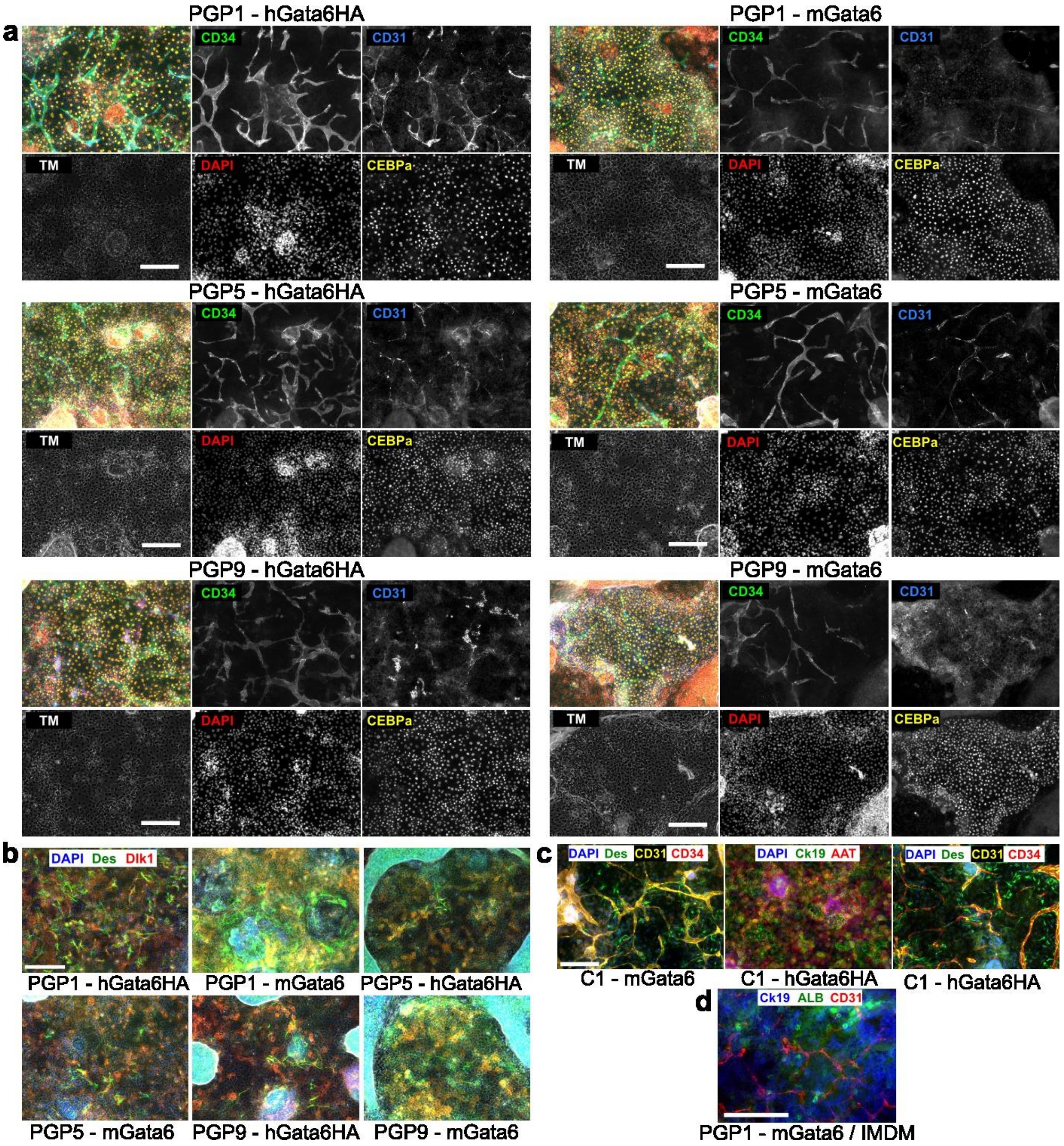

Supplementary Figure 19 | Generation of organoids with liver like phenotype in multiple human induced pluripotent cell lines. (a) Organoids were generated from PGP1, PGP5 and PGP9 cell lines and stained for CD34, CD31 (Endothelial-like cells), C/EBPα (hepatocyte-like cells) and DAPI (nucleus) on day 14. **(b)** PGP1, PGP5, PGP9 and C1 hiPSCs-derived liver like tissue fixed on day 14 and stained for DES (stellate-like cells), DLK1 (hepatocyte and stellate-like cells) and CD31 (endothelial-like cells). **(c)** Ck19 (hepatocyte and cholangiocyte-like cells), AAT (hepatocyte-like cells) and DAPI respectively. **(d)** Generation of ALB$^+$ hepatocyte-like cells and CD31$^+$ endothelial-like cells in plain IMDM medium on day 19. PGP1-hGata6HA images were also shown in Fig.5 a-c. Scale bars: 200 μm.

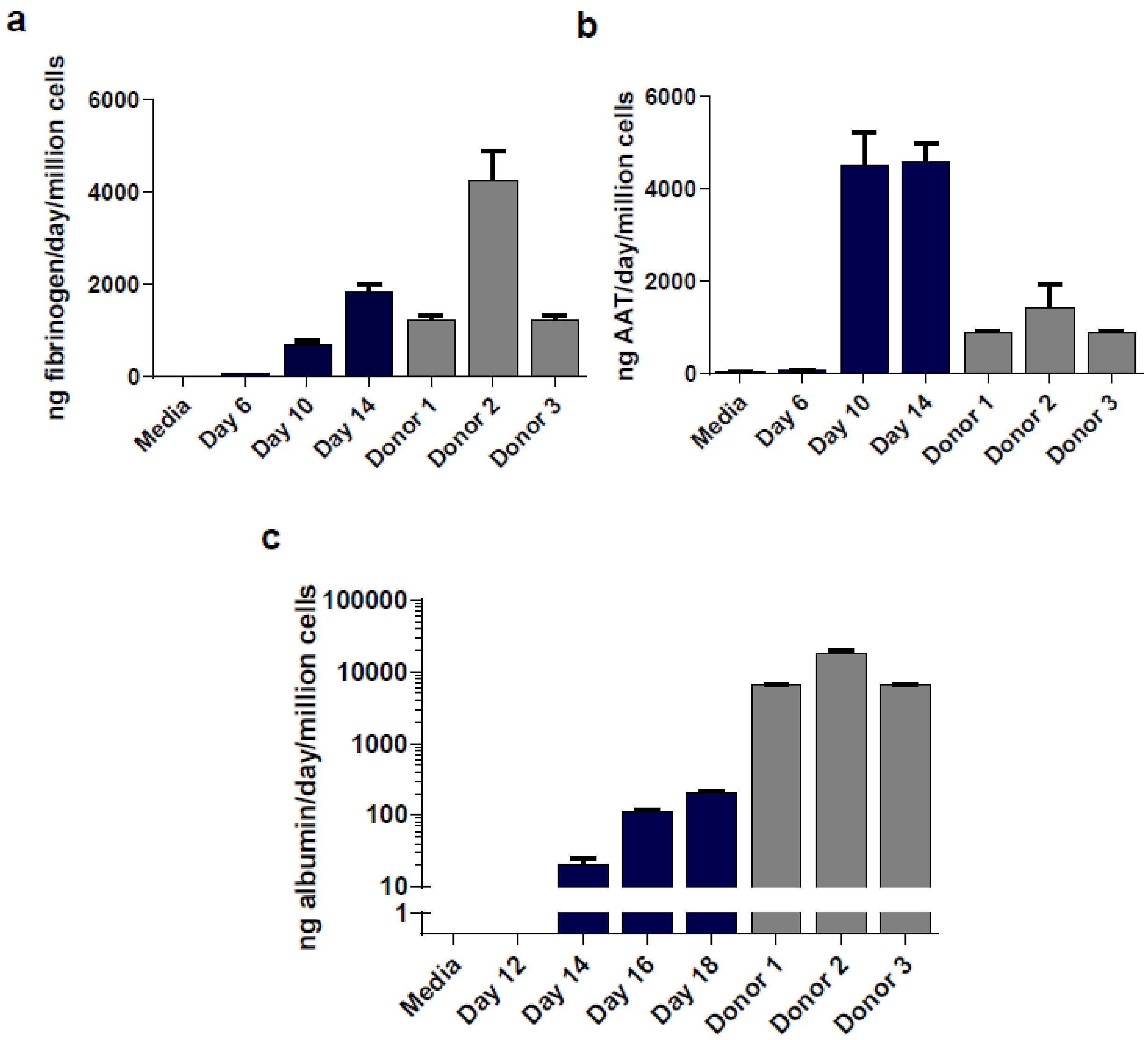

Supplementary Fig 20 | Comparison of metrics associated with hepatocyte functions. Comparison of metrics associated with hepatocyte functions among the developed tissues versus primary cryopreserved human hepatocytes from three different donors on day 3. Day 3 was chosen based on the maximum differentiation of primary hepatocytes in cultures, as they dedifferentiate in cultures observed by decreased values on day 7 *(data not shown)*. Dark blue bars represent protein secreted from organoids on different days of culture. Gray bars represent cryopreserved hepatocytes**.** **(a)** Secreted fibrinogen (ng/day/million cells). **(b)** Secreted AAT (ng/day/million cells). **(c)** Secreted Albumin (ng/day/million cells). For the hiPSC-derived organoids, values were normalized to the total number of HNF4α^{+} cells. Data are mean ± S.E.M, n=3 to 5 per group.

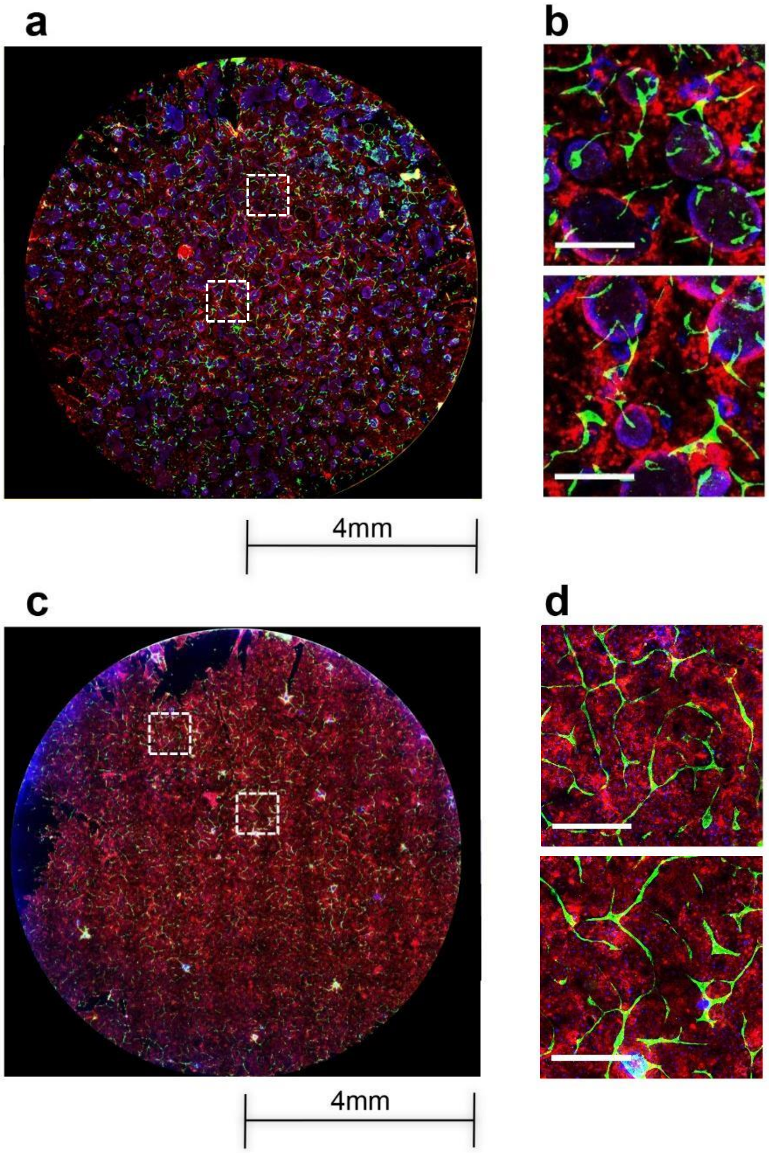

Supplementary Figure 21 | Macro-images of the developed liver bud like tissues. (a) Whole glass coverslip macro-image of unsorted *Gata6*-EGFP hiPSCs at day 14 of post-differentiation, stained for CD34 (green), AAT (red) and DNA (blue). Dotted white boxes identify the locations of two zoomed in images, shown in **(b)** 400µm scale- bars. The image shows AAT^{+} hepatocyte-like cells, CD34^{+} endothelial-like cells and CD34^{-}AAT^{-} non-transduced cell clusters of hiPSCs **(c)** Whole glass coverslip macro-image of sorted high EGFP expressing *Gata6*-EGFP hiPSCs, enriched by flow cytometry sorting and assessed at day 14 of post-differentiation by staining for CD34 (green), AAT (red) and DNA (blue). The flow cytometry gating is done based on the displayed information in Supplementary Figure 14a. Dotted white boxes identify locations of zoomed in images shown in **(d)** The image shows highly purified AAT^{+} CD34^{+} tissue without AAT^{-}, CD34^{-} cell clusters. Scale bars: 400µm.

Gene Set Name [# Genes (K)]	Description	# Genes in Overlap (k)	p-value	FDR q-value
KEGG_COMPLEMENT_AND_ COAGULATION_CASCADES [69]	Complement and coagulation cascades	20	0.00E+00	0.00E+00
KEGG_DRUG_METABOLISM_ CYTOCHROME_P450 [72]	Drug metabolism - cytochrome P450	11	3.31E-09	6.68E-07
BIOCARTA_AMI_PATHWAY [20]	Acute Myocardial Infarction	7	5.01E-09	6.73E-07
BIOCARTA_EXTRINSIC_PATHWAY [13]	Extrinsic Prothrombin Activation Pathway	6	8.97E-09	9.04E-07
BIOCARTA_FIBRINOLYSIS_PATHWAY [12]	Fibrinolysis Pathway	5	3.12E-07	2.51E-05
KEGG_METABOLISM_OF_XENOBIOTIC S_BY_CYTOCHROME_P450 [70]	Metabolism of xenobiotics by cytochrome P450	9	4.14E-07	2.71E-05
BIOCARTA_INTRINSIC_PATHWAY [23]	Intrinsic Prothrombin Activation Pathway	6	4.71E-07	2.71E-05
KEGG_STEROID_HORMONE _BIOSYNTHESIS [55]	Steroid hormone biosynthesis	8	6.97E-07	3.51E-05
KEGG_PENTOSE_AND_GLUCURONATE _INTERCONVERSIONS [28]	Pentose and glucuronate interconversions	6	1.66E-06	7.43E-05
KEGG_RETINOL_METABOLISM [64]	Retinol metabolism	8	2.28E-06	9.18E-05

Supplementary Table 1 | GSEA of genes exhibiting more than 4x upregulation between days 5 and 10. Prominently upregulated are pathways corresponding to liver metabolites, as for example thrombins and fibrinogens. The overlap with BIOCARTA_AMI_PATHWAY (Acute Myocardial Infarction) covers thrombin genes (produced by the liver) involved in blood clotting.

Gene Set Name [# Genes (K)]	Description	# Genes in Overlap (k)	p-value	FDR q-value
KEGG_COMPLEMENT_AND_ COAGULATION_CASCADES [69]	Complement and coagulation cascades	8	2.24E-08	9.01E-06
KEGG_CYTOKINE_CYTOKINE _RECEPTOR_INTERACTION [267]	Cytokine-cytokine receptor interaction	11	2.32E-06	4.67E-04
KEGG_NEUROACTIVE_LIGAND _RECEPTOR_INTERACTION [272]	Neuroactive ligand-receptor interaction	10	1.79E-05	2.41E-03
KEGG_PPAR_SIGNALING _PATHWAY [69]	PPAR signaling pathway	5	1.05E-04	1.06E-02
KEGG_TGF_BETA_SIGNALING _PATHWAY [86]	TGF-beta signaling pathway	5	2.97E-04	2.39E-02
KEGG_STEROID_HORMONE_ BIOSYNTHESIS [55]	Steroid hormone biosynthesis	4	5.22E-04	3.51E-02
KEGG_PURINE_METABOLISM [159]	Purine metabolism	6	7.60E-04	4.37E-02

Supplementary Table 2 | GSEA of genes exhibiting more than 4x upregulation between days 10 and 15. Prominently upregulated are pathways corresponding to liver metabolites, for example thrombins and fibrinogens, fat metabolism and steroid hormone biosynthesis.

Name	Oligo Sequence (3'-5')
oPG106	GAGCGGGATCCCCGGGTACCGGTCGCCACCATGTCTAGGCTGGACAAGAG
oPG107	GAGTCCGAATTCCCCGGGGAGCATGTCAAGGTCAAAATCG
oPG316a	CAAGCAATTGGAGGGCCGCGGCAGCCTGCTGACCTGCGGCGACGTGGAGGAAAAC
oPG316b	GACCTGCGGCGACGTGGAGGAAAACCCCGGCCCCATGAAAAAGCCTGAACTCACCGCGA
oPG317	GTAGAATTCGCGGCCGCTCAGTTAGCCTCCCCCATCTCCCGATC
oPG6370	TTTGGTCTCAAGGTTCGCCACCATGGCCTTGACTGACGGCGGC
oPG6371	AAAGGTCTCACTGCACTGGGACCCCGGGGCA
oPG6372	TTTGGTCTCAGCAGACCTGCTGGAGGACCTGTCCGAG
oPG6373	AAAGGTCTCACACGCGCTTCTGCGGCTTG
oPG6374	TTTGGTCTCACGTGCCTTCATCACGGCGGCTTGGATTG
oPG6375	AAAGGTCTCACCCATGGAGTTTCATGTAGAGTCCACAAG
oPG6376	TTTGGTCTCATGGGGTGCCCAGACCACTTGCTATGAAAAAAGAG
oPG6377	AAAGGTCTCACAGAGCAAGTCTTTGATTTATTTATGTTCTTAGG
oPG6378	TTTGGTCTCATCTGGTAATAGCAATAATTCCATTCCCATGACTCCAAC
oPG6379	AAAGGTCTCACCTGAGGCTGTAGGTTGTGTTGTGGGG
oPG6380	TTTGGTCTCACAGGGGCGGGTGCCCCGGTGATGACTG
oPG6381	AAAGGTCTCAAAGCTCAGGCCAGGGCCAGGGCGC
oPG630	GGGGACAAGTTTGTACAAAAAAGCAGGCTCGCCACCATGGCCTTGACTGACGGCGGC
oPG631	GGGGACCACTTTGTACAAGAAAGCTGGGTGGTATCAGGCCAGGGCCAGAGCAC
oPG6382	TTTGGTCTCAAGGTTCGCCACCATGGCCTTGACTG
oPG6383	AAAGGTCTCACCTCGGCCAGGGCCAGGGCGCAC
oPG6386	TTTGGTCTCACCTCCGCGCCCCGCAACCTC
oPG6385	AAAGGTCTCAGAGGCTCCAGGAAGGCGGGCACCCC

oPG_hG6_RegStartF	GGGGACAAGTTTGTACAAAAAAGCAGGCTTACCACCATGGCCTTGACTGACGGC
oPG6_RegTermR	GGGGACCACTTTGTACAAGAAAGCTGGGTATCAGGCGTAGTCAGGCACGTCGTAAGGATAGCCGGCCAGGGCCAGGGCGCAC
oPG_hGX-2A-EGFP_EGFP-fwd	TTTGGTCTCAGCGGCGACGTGGAGGAAAACCCCGGCCCCATGGTGAGCAAGGGCGAGGAG
oPG_hGX-2A-EGFP_EGFP-rev	AAAGGTCTCAAAGCTTACTTGTACAGCTCGTCCATGCC
oPG_hGX-2A-EGFP_G6-fwd	TTTGGTCTCAAGGTAGGTTCGCCACCATGGCCTTGAC
oPG_GX-2A-EGFP_G6-rev	AAAGGTCTCACCGCAGGTCAGCAGGCTGC
oPG5141	GGCAATTCCTCGAGAAAGCTAGGGCTCTTCATTAG
oPG5151	CTGCAGAAGAATTCTAGGACAAACGGAGGGAAATTAG
oPG1162	GGGGACAAGTTTGTACAAAAAAGCAGGCTCGCCACCATGGTGAGCAAGGGCGAGGAGCTG
oPG1163	GGGGACCACTTTGTACAAGAAAGCTGGGTGGTATTACTTGTACAGCTCGTCCA
oPG240	GGATCCTTAATTAACCAAACTGGATCTCTGCTGTCCC
oPG241	GTGGTGAATTCCGGCAATTCGATATCAAGCTTATCGATAATCAACC
oPG242	GAATTCTTAATTAATCAACTTTGTATAGAAAAGTTGAACGAGAAACG
oPG242	GAATCCCAATTGCCGGAATTCACCACTTTGTACAAGAAAGCTGAACGAG

Supplementary Table 3 | Oligos for plasmid constructions.

Supplementary Note 1 | Development of neural fold-like tissues

Alongside the mesendoderm-derived tissue, in clusters of cells that initially maintain high NANOG expression (Fig. 2a, b), the pluripotency and ectoderm marker OCT4 increases in intensity over the first five days (Supplementary Fig. 17a), and they subsequently acquire ectodermal markers between days 5 and 7 ($AP2^+$, $SOX10^+$). This process is followed by prominent fold-like outgrowths of cells expressing markers such as FOXG1, SIX6, PAX6, PAX7, DLX5 indicating differentiation to a neural lineage. Microarray analysis performed on day 15 post-induction on these outgrowths indicates an anterior/rostral fate with markers for neural crest and anterior neural folds (Supplementary Fig. 17b). One prominent marker is CYP26C1, a retinoic acid-catabolizing enzyme expressed first in the anterior part of the embryo and later in the rostral hindbrain next to the developing forebrain[1]. Immunostains reveal PAX6 and SIX6 at the top of these outgrowths in cup-like structures similar to optic cups (Supplementary Fig. 17 b)[2].

Supplementary References

1. Niederreither, K. & Dollé, P. Retinoic acid in development: towards an integrated view. *Nat. Rev. Genet.* **9,** 541–53 (2008).
2. Bharti, K. *et al.* A regulatory loop involving PAX6, MITF, and WNT signaling controls retinal pigment epithelium development. *PLoS Genet.* **8,** e1002757 (2012).
